# Genetic Manipulation and Bioreactor Culture of Plants as a Tool for Industry and Its Applications

**DOI:** 10.3390/molecules27030795

**Published:** 2022-01-25

**Authors:** Tomasz Kowalczyk, Anna Merecz-Sadowska, Laurent Picot, Irena Brčić Karačonji, Joanna Wieczfinska, Tomasz Śliwiński, Przemysław Sitarek

**Affiliations:** 1 Department of Molecular Biotechnology and Genetics, University of Lodz, 90-237 Lodz, Poland; 2 Department of Economic Informatics, University of Lodz, 90-214 Lodz, Poland; anna.merecz-sadowska@uni.lodz.pl; 3 Littoral Environnement et Sociétés UMRi CNRS 7266 LIENSs, La Rochelle Université, 17042 La Rochelle, France; Laurent.picot@univ-lr.fr; 4 Analytical Toxicology and Mineral Metabolism Unit, Institute for Medical Research and Occupational Health, 10000 Zagreb, Croatia; ibrcic@imi.hr; 5 Department of Immunopathology, Medical University of Lodz, 90-752 Lodz, Poland; joanna.wieczfinska@umed.lodz.pl; 6 Laboratory of Medical Genetics, Faculty of Biology and Environmental Protection, University of Lodz, 90-236 Lodz, Poland; tomasz.sliwinski@biol.uni.lodz.pl; 7 Department of Biology and Pharmaceutical Botany, Medical University of Lodz, 90-151 Lodz, Poland

**Keywords:** bioreactors, genetic manipulation, patents, recombinant proteins, secondary metabolites, transgenic plant cultures

## Abstract

In recent years, there has been a considerable increase in interest in the use of transgenic plants as sources of valuable secondary metabolites or recombinant proteins. This has been facilitated by the advent of genetic engineering technology with the possibility for direct modification of the expression of genes related to the biosynthesis of biologically active compounds. A wide range of research projects have yielded a number of efficient plant systems that produce specific secondary metabolites or recombinant proteins. Furthermore, the use of bioreactors allows production to be increased to industrial scales, which can quickly and cheaply deliver large amounts of material in a short time. The resulting plant production systems can function as small factories, and many of them that are targeted at a specific operation have been patented. This review paper summarizes the key research in the last ten years regarding the use of transgenic plants as small, green biofactories for the bioreactor-based production of secondary metabolites and recombinant proteins; it simultaneously examines the production of metabolites and recombinant proteins on an industrial scale and presents the current state of available patents in the field.

## 1. Introduction

The plant kingdom has always been closely linked with human society, serving as a source of both food and various remedies [1,2]. Thanks to the progressive development of science, the use of plants has grown in various industries [3,4]. In addition, the intensive development of phytochemistry has allowed for more in-depth determinations of the individual compounds contained in plant organs, i.e., leaves, roots, and stems [5,6,7]. Many molecules known to play an important role in the adaptation of plants to their environment also constitute an important source of pharmaceuticals [8,9]. In recent decades, this understanding of plant biotechnology has been dramatically expanded by the development of molecular biology. In vitro plant cultures have become an interesting research object due to the possibility of more precise modification [10]. The use of genetic engineering in particular has revealed multiple uses for plant cultures [11,12]. For example, by inserting appropriate genes into metabolic pathways, it is possible to dramatically increase the production of secondary metabolites [12,13].

The most popular secondary metabolite production systems are hairy roots induced by *Rhizobium rhizogenes*, as these offer good genetic stability together with the possibility to obtain a large amount of material in a short time [14]. This solution has become frequently used in the pharmaceutical industry for the production of valuable compounds such as taxol, vinblastine, and camptothecin [15,16]. However, that system is not as useful due to the need to culture in dark [17]. Another interesting approach is the use of plants to produce high value recombinant proteins for industrial and clinical applications; this has become a promising alternative to conventional expression systems (bacteria, yeast, or animal cells). However, it should be remembered that the production of secondary metabolites as well as recombinant or modified proteins is a complex procedure that requires an interdisciplinary effort covering a wide range of scientific and technological disciplines [18,19].

Both presented systems offer several advantages over conventional strategies in terms of the safety and availability of material, which shows that transgenic plants function as small factories that produce secondary metabolites or recombinant proteins [20]. However, to overcome their limitations, which are mainly related to the scale of production, plant material is often cultivated in bioreactors, these being engineering systems capable of supporting optimal conditions for aerobic or anaerobic biochemical processes. Such culture methods are characterized by stability, ease of use, increased nutrient uptake ability, time, and cost efficiency, as well as large biomass yields; as such, bioreactor culture is regarded as a suitable alternative to conventional methods of plant tissue culture on an industrial scale [21,22].

Creating such an efficiently operating production system with a specific material or under strictly-defined conditions may result in a potential patentable application [23,24]. As such, there are many patents of this type relating to the culture of transgenic material in bioreactors for both secondary metabolites and recombinant proteins [25].

The main task of this review is to familiarize readers with the latest strategies used when working with in vitro plant cultures to increase their productivity in terms of metabolites and proteins with potential medical applications. In this review, we focus on the use of bioreactors in the cultivation of various plant cultures in vitro. The applications of the various types of bioreactors for the cultivation of plant material, examples of the manipulation of metabolic pathways for increased productivity of secondary metabolites, obtaining transgenic plant cultures in vitro for the production of recombinant proteins, as well as a review of patented bioreactors that can be used to cultivate plant cells and tissues under controlled conditions are presented here. This work may be an introductory guide for researchers planning to conduct research in green biotechnology using a variety of approaches to obtain desired products.

## 2. Criteria for the Selection of Experimental Articles in the Analyzed Subject

This paper reviews the current state of knowledge regarding in vitro transgenic plant cultures in bioreactors aimed at the production of secondary metabolites and recombinant proteins based on papers published in the last ten years (2010–2021).

**Figure 1:**
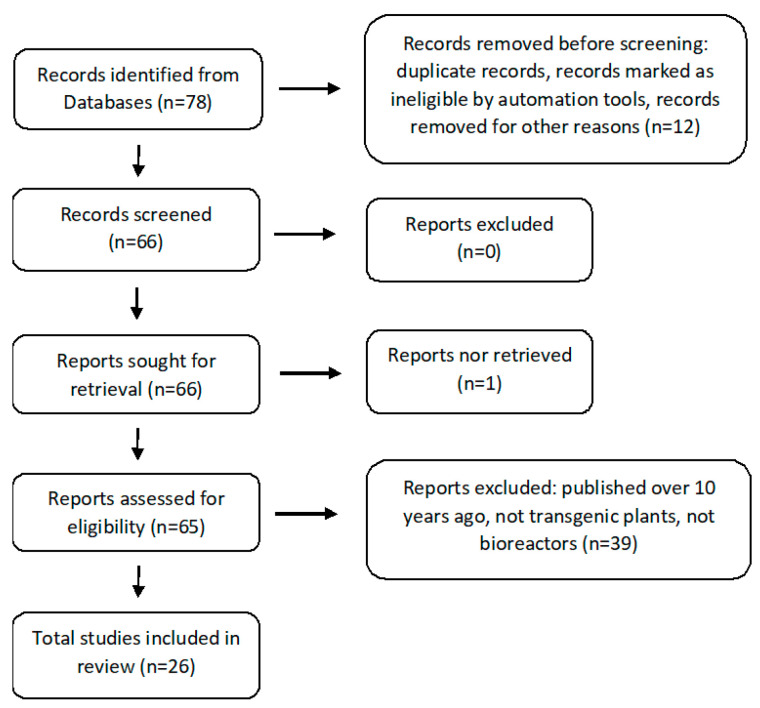
PRISMA flow diagram demonstrating screening method for article [26].

The studies were selected from the electronic databases PubMed/MEDLINE, Scopus, Web of Science, and Google Scholar. The following search terms were used: transgenic plants, secondary metabolites, recombinant proteins, and bioreactors. The search included published experimental studies reporting about various in vitro plant transgenic cultures and focused on the production of various secondary metabolites and recombinant proteins grown in bioreactors on an industrial scale as well as any patents relating to these studies. Papers reporting on articles published in languages other than English, those with only an abstract or lacking full text access, or published over ten years ago were excluded. Each selected document was analyzed and the following data were extracted and presented in a table: scientific species names, plant material used for transformation, vector used f+ or transformation, type of metabolite, type of recombinant protein, bioreactor capacity, and final effect (Figure 1). The main text includes various studies, including examples of transgenic cultures producing increased levels of secondary metabolites and recombinant proteins.

## 3. Transgenic Plants—Brief History and Potential Industrial Use

Transgenic plants are desirable to industry. Although the first organisms to be genetically modified were bacteria, their potential commercial value was quite limited and were of interest only as an initial model [27,28]. However, the development of transgenic plants promised a completely different scale of production. Commercial interest in biotechnology grew rapidly from the late 1970s to the early 1980s [29]. In 2019, 190.4 million hectares of biotech crops were planted by 17 million farmers in 29 countries, an increase of more than 100-fold since the initial planting of 1.7 million hectares in 1996 [30].

As such, biotech crops are considered to be the most rapidly adopted culture technology in the history of modern agriculture. As of 2019, the United States is home to the largest area of genetically-modified crops worldwide at 71.5 million hectares, followed by Brazil with a little over 52.8 million hectares [31]. The most commonly-grown biotech crops in 2019 were soybeans, corn, cotton, and canola [32]. While there was a 4% reduction in biotech soybean planting, the high adoption rate of 48% of the world’s biotech crops, or 91.9 million hectares, was maintained [31]. This acreage accounted for 74% of the total soybean production in the world in 2019 [33].

Generally, transgenic plants are defined as those whose DNA is modified by genetic engineering techniques. Although they were arguably first implemented 10,000 years ago in Southwest Asia, where humans first grew plants through artificial selection and selective cultures [34,35], the first modern transgenic plants were developed in the early 1980s in an experiment where bacteria and yeast genes were cloned into the Ti plasmid and then used to create a plant containing those new sequences [36]. The results of the experiment and the transgenic plant were published in April 1983 [37].

Transgenic plants contain an artificially inserted gene or genes. These sequences are known as transgenes; they may originate from an unrelated plant or from an entirely different species. The purpose of introducing a combination of genes into a plant is to make it as useful and productive as possible, or for the addition of completely new features [38,39]. The development of molecular plant breeding has resulted in technological advances in transformation methods, gene expression regulation, protein sorting and accumulation, and the use of various crops as production platforms [40].

One of the most important factors in the plant genetic transformation procedure is the type of explant. For example, the use of different sources of plant cells or tissues (e.g., seeds, roots, shoots, leaves, and shoot or root apical meristems) results in different degrees of callus induction, and hence different levels of plant regeneration and genetic transformation efficiency [41,42]. Many plants (such as soybean, corn, wheat, tomato, rice, etc.) have been genetically modified to achieve higher yields, stress tolerance, and enhanced production of high-value compounds such as recombinant proteins, antibodies, enzymes, and vaccines. This makes them a promising alternative to conventional bioproduction systems, such as bacteria, yeast, and cultured insect and animal cells [43]. An example of such a positive modification was the nutritionally valuable “golden rice” and “golden bananas”, which had elevated levels of β-carotene—a precursor of vitamin A [27,44,45]. Another example is tomato fruit producing squalene, phytosterols, α-tocopherol, and carotenoids [46].

One of the best plant-based alternatives for the production of recombinant proteins are plant-cell suspensions; this is a robust system involving a simple purification procedure and easy downstream processing [47]. In turn, the transformed roots formed by infection with *R. rhizogenes* have become an alternative for the production of secondary metabolites and have been the subject of intensive research for several decades. They are characterized not only by genetic stability but also by a fast growth rate, which translates into obtaining a large biomass in a short time. In addition, this process allows us to obtain biologically valuable compounds without destroying the entire plant and its habitat, which is extremely important in the era of environmental protection [16,48,49]. Transgenic plants can also represent a valuable industrial-scale source of bioactive secondary metabolites used in many fields of pharmacology or medicine [50].

Modern biotechnology has led to a revival of interest in obtaining new active therapeutic compounds from plant sources. In turn, metabolic engineering has proven to be an effective tool for manipulating biosynthetic pathways by increasing or decreasing selected gene expression [51,52]. The advantage of transgenic plants over other expression systems makes them an attractive alternative for producing secondary metabolites or recombinant proteins [53,54,55].

The main advantages of transgenic plants are increased yields [56], resistance to diseases and pests, and the possibility for growth under stress conditions [57], including drought [58,59,60], heat [61], and frost [62]. In addition, foods can be given increased resistance for transport over long distances: the plants are harvested early and can ripen during transport, which ensures a longer shelf life. Even with extended shipping and storage periods, the product reaches its destination without spoiling. In addition, the producers of these crops promote them as the second “green revolution” in a world with an ever-increasing population [63]. In the pharmaceutical sphere, transgenic cultures are a platform for obtaining recombinant proteins and valuable secondary metabolites [50].

Unfortunately, transgenic plant production also has disadvantages. It has been suggested that the antibiotic resistance genes present in these crops result in the wider development of antibiotic resistance [62]. Another problem may be the emergence of super-pests and the loss of biodiversity if these plants are used in an uncontrolled manner [27,64]. This situation is of course not possible in the case of closed and controlled in vitro cultures. Currently, transgenic plants are rationally used and have found versatile applications, particularly as bioreactors for the production of proteins and various biopharmaceuticals, such as erythropoietin—used in the treatment of anemia—and insulin—used in the treatment of diabetes [42,65].

The issues related to the genetic modification of organisms described in the article obviously touch upon social and ethical problems. Arguments are often raised here about the potential threats to human health and the natural environment, traditional agricultural practices, or even corporate dominance from large biotechnology concerns. It is worth emphasizing that, when assessing the possible benefits and potential threats, each case should be assessed individually and holistically. It is important to bear in mind the significant benefits of genetically modified plants, such as pest resistance, increased levels of nutrients, and improved productivity. The arguments presented above are usually raised in the case of field cultivation of genetically modified crops. For the examples described in our article, many of these arguments are not justified because these cultures are kept strictly controlled and isolated from the environment in in vitro conditions. In such systems, the possibility of a significant increase in the productivity or synthesis of completely new compounds with a wide range of applications, including new and more effective therapies for many human diseases, is the best rationale for many skeptics. The biggest mistake of the scientific community in this field would be not using the tools available today that lead to the development of many valuable technologies in the service of humanity.

## 4. Plant Tissue and Organ Cultures in Bioreactors

Products containing ingredients of plant origin have been indispensable elements of food products, medicines, dietary supplements, or various types of cosmetics for centuries [66,67,68,69,70]. It is well known that the biologically active compounds contained in plant tissues have beneficial effects on health and beauty. As such, there is growing interest in plant products as sources of extremely attractive ingredients in many products. However, a greater awareness of climate change and the desire to reduce the use of chemical plant protection products or to conserve water resources has forced the search for alternative sources of phytochemicals, such as those from ginseng or aloe. One attractive course of action may be the culture of plants in strictly controlled and optimal in vitro conditions.

Another important advantage of plant culture is the possibility of increasing the scale of the entire culture process, and a number of new culture systems for plant in vitro cultures have been developed [10,70,71,72,73,74]. Combining these culture strategies with the use of special procedures, such as elicitation with biotic or abiotic elicitors, allows for the large-scale production of the target biologically active compound [75]. As such compounds are typically complex molecules, their chemical synthesis is often very complicated and unprofitable. Given this, plant cells are now enjoying growing popularity as miniature factories. Since the times of Gottlieb Haberlandt—who in 1902 initiated the use of plant cell and tissue cultures—describing the process of plant regeneration from callus cultures, there has been a revolution related to the possibilities of their laboratory and industrial application. However, the turning point in bioreactor culture occurred in 1956 when Routien and Nickell [76], in cooperation with Pfizer Inc., obtained the first patent for a large-scale in vitro culture of plant cells for secondary metabolites. Subsequently, the techniques of culturing plant cells and organs were improved through the mid-1950s, with a significant achievement being the development of a complete medium for the culture of plant tissues by Murashige and Skoog [77]. Since then, our understanding of plant cells has changed significantly, and the current technological possibilities allow even the most demanding cells and tissues to be cultivated, thus allowing the efficient production of valuable biologically active compounds. Many technical solutions have been developed to optimize the culture of plant material depending on its origin and specificity. One such example is the bioreactor, most simply defined as a vessel in which a biological reaction or change takes place, and which provides optimal conditions for the culture of cells, tissues, or whole plants [21,78]. Such bioreactors can be very structurally diverse. Laboratory conditions most often require the use of reactors with a smaller volume and simpler structure, these are often tightly closed vessels with a stirrer, sprinkler, or aeration nozzle, while the industrial ones are complex devices in which most parameters are controlled digitally [79,80]; they are most often equipped with a microprocessor control unit to monitor the pH, temperature, and density of the cultured cells. The selection of the bioreactor and the process itself depend on the method of production, i.e., secretion into the culture medium or accumulation in biomass. The most common process modes used in such cultures are batch culture, fed batch culture, rapid feed batch culture, two-stage batch culture, and continuous culture. As the optimum conditions for the growth of different types of plant cells or tissues vary considerably, choosing the right bioreactor is not always easy. The most common types of bioreactors are presented below.

### 4.1. Bubble Column Bioreactors

This is a type of gas–liquid dispersion reactor. This is the simplest type of bioreactor and consists of a cylindrical culture vessel and a sparger mounted on the bottom. The culture medium in this type of bioreactor is mixed by air bubbles released from the bottom of the vessel. As mechanical mixing is not used, this type of bioreactor is very often used for the culture of plant tissue cultures. In the case of plant material, this type of bioreactor’s aeration significantly reduces the exposure of the delicate cells to shear stress while ensuring thorough mixing of the biomass to be grown. Of course, this type of bioreactor has many advantages related to its simple construction, potential for increased scale, and low construction costs. The main disadvantages include a relatively low degree of mixing of the medium or high foaming [81].

### 4.2. Stirred Tank Bioreactors

Another extremely popular type of bioreactor for growing plant material is the stirred tank reactor. These devices have a special stirrer that helps to maintain appropriate conditions throughout the entire volume of the culture medium. It is worth noting that stirred tank bioreactors perform better in the case of higher viscosity cultures due to the minimization of microzones with poor mixing of the medium. In the case of plant material, the mixing speed is usually low due to the high shear forces. This phenomenon, in the case of hairy roots, often leads to the appearance of unfavorable callus tissue. For this reason, technical solutions are used to separate the stirrer from the cultured cells [81,82] or by using flat-blade turbine impellers. Such bioreactors were used in the cultivation of *Vitis labrusca* cell suspension cultures [83].

### 4.3. Nutrient Mist or Sprinkle Bioreactors

In nutrient mist bioreactors, the cultured material is immobilized in a growth chamber, while the culture medium is delivered to it in the form of an aerosol. This solution provides good access to both oxygen and nutrients for the growing plant material. In addition, the occurrence of dangerous shear forces is eliminated, which allows the culture of delicate plant tissues without the need for additional protection. A similar culture strategy is also provided by nutrient sprinkle bioreactors, in which the growing tissue can be immobilized in a mesh, basket, or other matrix, most often made of stainless steel. The culture medium is circulated with a peristaltic pump and the plant material is sprayed periodically or continuously to provide nutrients. Depending on the needs, the spraying nozzle is located above or below the plant tissue, or two nozzles are used. A very important parameter in this bioreactor is to maintain the appropriate diameter of the formed droplets connected with the flow without excessive fluid retention on the surface of the growing tissue. In addition, the use of this type of bioreactor for the cultivation of high-density branched roots significantly contributes to the achievement of good results. Large-volume cultures may be a problem here, which generates limitations related to the preparation of a sufficiently large column. The necessity to ensure an optimal distribution of the culture medium necessitates a significant increase in the size of the entire device [82,84].

### 4.4. Wave-Mixed Bioreactors

Wave-stirred bioreactors consist of a pillow-like culture bag that relies on the swaying motion of the platform to produce a bubble-free agitation and aeration wave. The swing angle and speed must be carefully adjusted to meet the required homogenization of the culture and gas transfer through the headspace of the bag solution. Wave-stirred bioreactors also monitor and control temperature, pH, and dissolved oxygen (DO). Wave bioreactors are a specific type of reactor in which the movement of the culture medium is forced by undulations caused by the movement of a culture bag. The bag can be only partly filled with culture medium (up to 50%). Appropriate selection of the movement parameters (speed and angle of inclination) allows the culture process to be optimized. This continuous movement of the bag allows for thorough mixing of the culture and efficient gas exchange. Most such reactors use disposable bags that are disposed of after culture, thus reducing the production costs. This type of bioreactor can be used to grow plant suspension cultures or hairy roots [81,82,85,86,87].

### 4.5. Temporary Immersion System

TIS (Temporary immersion system) are automated platforms for controlled short-time contact of the plant explants with a liquid medium in an aseptic environment, which could be a particularly interesting option in terms of *Rhizobium*-mediated genetic transformation techniques. This type of reactor allows the culture of various plant tissues and organs by periodically immersing them in the culture medium. This type of reactor allows for a good balance between the access of oxygen and nutrients, thus obtaining good biomass growth. Moreover, optimization of the culture conditions is easily accomplished by manipulating the frequency and time of immersion in a liquid medium. Most importantly, the tissues and organs are not exposed to the destructive effects of shear forces occurring in the other types of reactors. The top vessel is the rearing chamber; it is equipped with a water jacket for precise temperature control and an integrated UV light source, which is mounted on the top cover. The plant material is supported by a stainless-steel screen installed inside the growing chamber. The bottom vessel is the nutrient tank; it is designed with two external ports—one on the top end for air supply and one at the bottom end for media loading and sampling.

The above examples are intended as a brief introduction to the most commonly used reactors for the in vitro culture of plant material. It is important to note that each system should be set up individually depending on the cultured cells, tissues, organs, or whole plants. When used on an industrial scale, many approaches are based on combinations of different strategies that are intended to ensure the maximum productivity of secondary metabolites or recombinant proteins [23,88].

**Figure 2:**
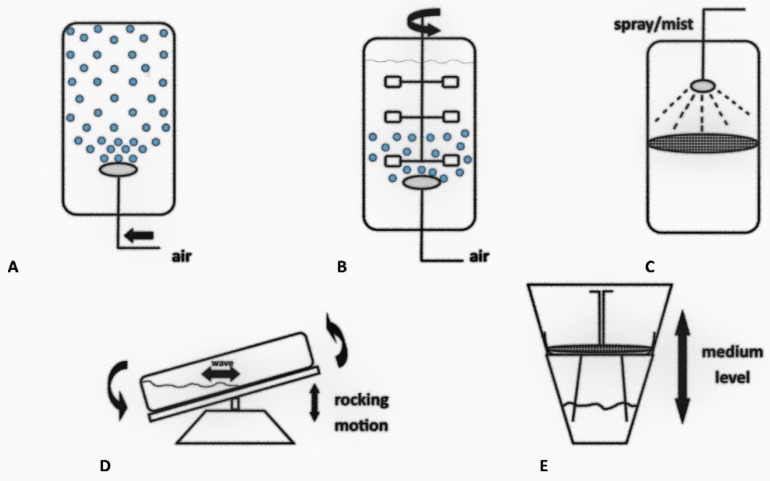
The types of bioreactors most often used for the cultivation of plant cultures in vitro. (**A**) Bubble column bioreactor, (**B**) Stirred tank bioreactor, (**C**) Nutrient mist or sprinkle bioreactor, (**D**) Wave-mixed bioreactor, (**E**) Temporary immersion system.

The types of bioreactors described previously are shown in Figure 2.

## 5. Transgenic Plants Manipulated in Metabolic Pathways as a Source of Bioactive Secondary Metabolites Grown in a Bioreactor

The World Health Organization defines any plant that contains a substance that can be used for therapeutic purposes or that is a precursor to a new, semi-synthetic pharmaceutical as a medicinal plant [89]. Over the course of evolution, plants gradually gained the ability to synthesize various types of secondary metabolites with valuable medicinal properties [90]. Secondary metabolites are useful natural products synthesized by the secondary metabolism of plants. The production of some secondary metabolites is associated with the induction of morphological differentiation and maturation in some cells during plant growth [53]. Plant secondary metabolites are most often classified according to their biosynthetic pathways. Individual compounds are synthesized via a variety of enzyme-catalyzed reactions using simple building blocks. There are several major biosynthetic pathways in plants, including the mevalonate pathway (joining three molecules of Acetyl-CoA) and the non-mevalonate pathway—which both produce isopentenyl diphosphate (IPP) and dimethylallyl diphosphate (DMAPP) (terpenes)— the shikimic acid (phenylpropanoids) pathways, the 2-C-methyl-d-erythritol-4-phosphate (quinones) pathway, the amino acid pathway (alkaloids), the acetate-malonate pathway (fatty acids, phenols, and quinones), and the complex pathway (flavonoids) [13,91].

Generally, four groups of large molecules are recognized: phenols, steroids, terpenes, and alkaloids. While phenols are involved in the synthesis of lignin and are characteristic of all higher plants occurring in nature, other bioactive compounds, such as alkaloids, are sparsely distributed in the plant kingdom and are much more specific to a particular genus and species. This narrower distribution of secondary compounds forms the basis of chemotaxonomy and chemical ecology [92,93].

Due to their high biological activity, plant secondary metabolites have been used in traditional and folk medicine for the prevention of many diseases for centuries. Nowadays, they are used on an industrial scale for the production of pharmaceuticals, cosmetics, fine chemicals, and, recently, dietary supplements and nutraceuticals [94,95]. In the era of the advancing chemical industry and the use of artificial synthetic compounds, plants have once again become the subject of intensive research and are currently experiencing a renaissance. This is mainly related to the acquisition of compounds with potentially new biological properties and natural origins [96,97]. In particular, the interest in the possibility of obtaining and increasing the production of bioactive secondary metabolites in plants has resulted in the development of a new field of science, plant biotechnology. Biotechnological production of valuable secondary metabolites in cultures of plant cells or organs is an attractive alternative to the extraction of total plant material [98,99]. These approaches have had varied commercial successes; this is largely related to the selection of high-performance, stable cultures and the use of the poorly understood mechanisms of regulation and synthesis of these compounds in metabolic pathways [100]. However, many studies have confirmed the increased production of secondary metabolites from medicinal plants based on a range of biotechnological strategies [101,102]. Some of these include screening for high-throughput cell lines, media modification, precursor feeding, elicitation with biotic or abiotic elicitors, large-scale bioreactor culture, hairy root culture, plant cell immobilization, biotransformation, metabolic engineering, etc. [94,103,104].

One dynamically developing branch of plant biotechnology in recent decades is metabolic engineering, which has yielded increases in the production of secondary metabolites in plant cultures in vitro by interference in biosynthetic pathways [105]. The main goal of metabolic engineering is to improve cellular activity by manipulating the cellular enzymatic, transport, and regulatory functions using recombinant DNA technology. This approach typically involves the identification of enzyme-limiting activities by the successful elucidation of the pathway and associated metabolites; these limiting steps can be improved by the appropriate application of genetic transformation. The vast majority of the strategies presented so far rely on the introduction of genes isolated from more efficient organisms, promoters enhancing target gene expression, or antisense and co-suppression techniques to achieve the desired properties [106,107,108,109]. The most common example of such manipulation is *Rhizobium rhizogenes*-mediated transformation, which has the advantage of being able to transfer any foreign gene of interest that is placed in a binary vector into the plant genome. A number of other alternative transformation methods also exist, such as microprojectile bombardment, direct protoplast transformation, microinjection, pollen-tube pathway, or liposome-mediated transformation.

It is also possible to selectively alter certain secondary metabolites of plants or cause their secretion by introducing genes that encode enzymes that catalyze certain hydroxylation, methylation, and glycosylation reactions [16,110]. An example of a gene for secondary metabolism is the 6-β hydroxylase gene, which codes for a tropane alkaloid used in medicine and was introduced into the hairy roots of *Scopolia parviflora* through the *R. rhizogenes* binary vector system; the modified roots showed 8.12 mg greater scopolamine per g dry weight [111]. Scopolamine that belongs to the anticholinergic class of drugs is used to relieve post-operative nausea, vomiting, and motion sickness [112]. In turn, Kowalczyk et al. showed that *Senna obtusifolia* transgenic hairy roots with an overexpression of the *PgSS1* gene from *Panax ginseng* contain a higher level of betulinic acid, a triterpenoid saponin, than roots without the construct [113]. Betulinic acid has various biological properties, including antioxidant, anti-inflammatory, and anti-cancer. The data indicate that their anti-tumour effects are triggered by the induction of apoptosis via the mitochondrial pathways in cancer cells [114]. Shim et al. revealed that the overexpression of the ginsenoside’s biosynthetic pathway key gene *PgSQS1*, which can upregulate the expression of squalene epoxidase, β-amyrin synthase, and cycloartenol synthase, resulted in a twofold increase of phytosterols and a 1.6- to 3-fold increase of total ginsenosides, a group of glycosylated triterpenes, in transgenic ginseng adventitious root cultures [115]. Ginsenosides also act as antioxidation and anti-inflammatory factors and have beneficial effects on cardiac and vascular diseases [116]. In turn, Jian et al. noted that the expression of the tomato regulatory gene *SlMYB75*, an MYB-type transcription factor, promotes anthocyanin accumulation in tomato fruits [117]. Overproducing of anthocyanins can be an effective way to extend a tomato’s shelf life [118]. Elsewhere, Sitarek et al. showed a significant increase in the production of phenolic acids, including neochlorogenic acid, chlorogenic acid, caffeic acid, *p*-coumaric acid, and ferulic acid, in the transgenic roots of *Leonurus sibiricus* through the overexpression of the AtPAP1 transcriptional factor [119]. Plant-derived phenolic acids are considered to be natural antioxidants with potential health benefits [120]. Sun et al. showed that overexpression of the octadecanoid responsive *Catharanthus AP2*-domain protein (ORCA3) and strictosidine glucosidase in the hairy roots of *Catharanthus roseus* increased the production of terpenoid indole alkaloids by 47%, including serpentine, ajmalicine, catharanthine, tabersonine, lochnericine, and hörhammericine—all of which exhibit interesting pharmaceutical activities such as anticancer, antimalarial, and antiarrhythmic functions [121]. Esposito et al. demonstrated that the transformed roots of *Taxus baccata* with the overexpression of the *TXS* gene showed 265% greater diterpene and taxane production after MeJA elicitor treatment [122]. The taxanes, paclitaxel and docetaxel, act as anticancer agents by stabilizing the microtubules during cell division [123].

Clustered regularly interspaced short palindromic repeats (CRISPR) along with the CRISPR-associated proteins (Cas) system that was found in various bacteria serves as a defense mechanism against viruses. The potency of the technology enables the ability to target any sequence throughout a cell. Thus, this can be a genome editing system that may regulate secondary metabolism in plants [124]. The data showed that the contents of phenolic acids, including rosmarinic acid and lithospermic acid B, as well as tanshione synthesis were decreased in the knocked out rosmarinic acid synthase gene [125] and diterpene synthase gene [126] lines of *Salvia miltiorrhiza,* respectively. *Arabidopsis thaliana* and its ubiquitin-protein ligase (HOS1) mutants are characterized by changes in phytoalexins synthesis [127].

In addition, bioreactor cultures may also increase the secondary metabolite production by increasing biomass, thus creating a fairly cheap system for the production of active compounds. Of course, it should be remembered that the cultivation of cells, tissues, and plant organs in a bioreactor also has some limitations, despite the huge advances in bioprocess engineering. One of them is the time-consuming and labor-intensive procedures related to the aging, inoculation, and cleaning of the entire system before and after cultivation. Other disadvantages include the possibility of occasional intensive foaming, the presence of shear forces in some types of reactors, or the provision of uniform culture conditions throughout the volume without unduly limiting the viability of the plant cells. Fortunately, the vast majority of these limitations can be overcome with an optimal bioreactor design and appropriate adjustment of the key parameters of the culture system [78].

Table 1 presents the selected examples of secondary metabolites obtained from transgenic plants that were cultured in bioreactors; including caffeic acid (CA) and chlorogenic acid (CHA) from *Leonurus sibiricus;* vincamine from *Vinca minor;* protopanaxadiol (PPD)-type ginsenosides from *Nicotiana tabacum (N. tabacum);* geraniol from *N. tabacum;* swertiamarin, gentiopicroside, and sweroside from *Centaurium maritimum;* β-elemene from *Curcumae zedoariae;* and betulinic acid from *Senna obtusifolia*. The details about the vectors or genetic construct elements as well as the culture’s conditions in bioreactors are also included.

The presented examples showed that plant systems are good models for the production of secondary metabolites.

According to Table 1, various classes of secondary metabolites are produced by transgenic plants cultured in bioreactors. The exploring of their biological activity show that all of them exert anti-cancer properties.

PPD is a triterpenoid saponin that belonged to ginsenosides, the major active component of ginseng. This compound could affect cell cycle distribution and pro-apoptotic signaling. Therefore, it is proposed that this compound might be a potent addition to the current chemotherapeutic strategy against cancer [135].

CA and CHA belong to the phenolic compounds. They possess a vulnerable activity for neutralizing reactive oxide species. Oxidative stress may activate a number of transcription factors, which lead to the differential expression of some of the genes involved in inflammatory pathways. Therefore, polyphenols have been proposed to be useful as an adjuvant therapy for their anti-inflammatory effects and may be helpful for the development of future antioxidant therapeutics and new anti-inflammatory drugs [136]. In addition, phenolic compounds have anti-cancer properties via modulating key processes such as oncogenic transformation of normal cells, growth and development of tumors, and angiogenesis and metastasis [137].

Betulinic acid is a pentacyclic triterpenoid with a wide range of biological properties, such as anti-inflammatory and anti-cancer. The activity of betulinic acid has been linked to the induction of the intrinsic pathway of apoptosis in cancer cells. In contrast to cancer cells, normal cells and tissues are relatively resistant to that compound. Given this, this compound seems to be a promising experimental cancer therapeutic [138,139].

Another compound with anti-cancer activity is geraniol, a monoterpene alcohol. The data indicate the preventive effects of geraniol on different types of cancers, including lung, colon, breast, prostate, pancreatic, and hepatic cancer. This compound has a pleiotropic effect on cancer hallmarks, including sustaining proliferative signaling, evading growth suppressors, enabling replicative immortality, tumor-promoting inflammation, inducing angiogenesis, genome instability and mutation, resisting cell death, and deregulating cellular energetics. Moreover, geraniol sensitizes the tumor cells to selected chemotherapy agents [140].

Swertiamarin is a seco-iridoid glycoside that meets all five of Lipinski’s rules for drug-like properties. Therefore, it possesses many beneficial pharmacological properties, including hepatoprotective, analgesic, anti-inflammatory, antiarthritis, antidiabetic, antioxidant, neuroprotective, and gastroprotective activities. Moreover, there have also been recently reports of the anticancer activity of swertiamarin against different cancer cell lines [141].

β-elemene is a sesquiterpene with potential anti-inflammatory and anti-cancer properties. This compound plays a role in macrophage infiltration and M2 polarization, regulates the transcription factors NF-κB and STAT3 to alter inflammation, and tumorigenesis and development. In addition, β-elemene modulates different inflammatory factors (such as TNF-α, IFN, TGF-β, and IL-6/10) as well as oxidative stress in vivo and in vitro [142]. Apart from enhancing the immune system, β-elemene exerts its effects in cancer cells by inhibiting cell proliferation, arresting the cell cycle, inducing cell apoptosis, exerting anti-angiogenesis and anti-metastasis effects, and reversing multiple-drug resistance [143].

Vincamine is a monoterpenoid indole alkaloid with a vasodilatory property. Studies indicate that vincamine increases the regional cerebral blood flow [144]. Moreover, it was found that vincamine stimulated apoptosis and lowered mitochondrial membrane potential. Vincamine was also found to neutralize hydroxyl free radicals and deplete iron ions in cancer cells [145].

**Figure 3:**
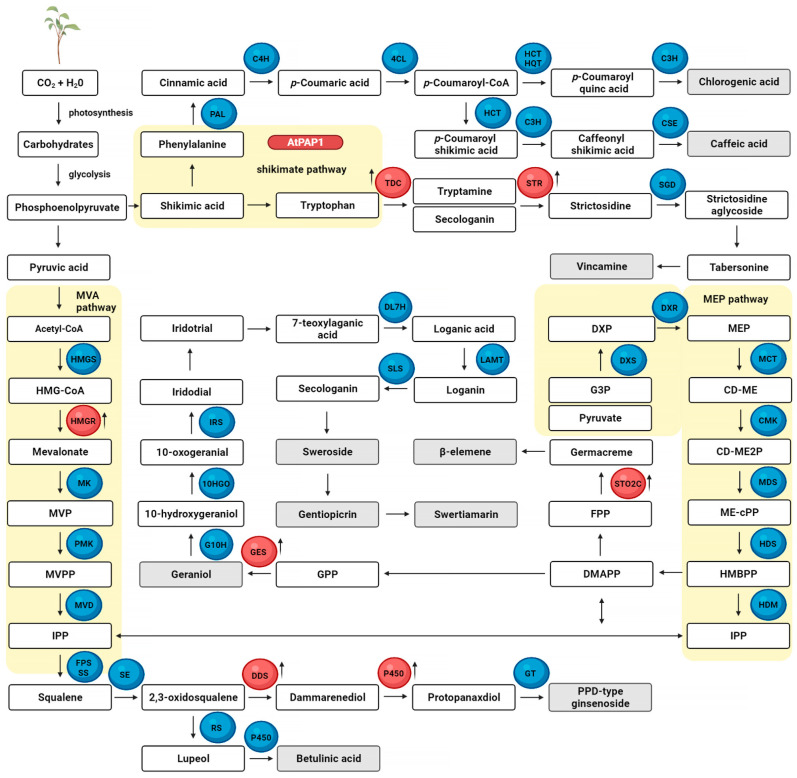
Schematic of the selected secondary metabolite biosynthesis pathway of transgenic plants with incorporated genes. The enzymes marked in red are expressed by plant species presented in Table 1.

Selected metabolic pathways with research examples presented in Table 1 are given in Figure 3. The up arrow indicates the increased production of those compounds.

Abbreviations: 10HGO: geraniol 10-hydroxylase; 4CL: 4-coumarate-CoA ligase; C3H: p-coumarate 3′-hydroxylase; C4H: cinnamate-4-hydroxylase; CMK: 4-diphosphocytidyl-2-C-methyl-D-erythritol kinase; CSE: caffeonyl shikimate esterase; DDS: dammarenediol synthase; DL7H: 7-deoxyloganic acid 7-hydroxylase; DMAPP: dimethylallyl diphosphate; DXR: 1-deoxy-d-xylulose-5-phosphate reductoisomerase; DXS: 1-deoxy-d-xylulose-5-phosphate synthase; FPP: farnesyl diphosphate; FPS: farnesyl diphosphate synthase; G10H: geraniol 10-hydroxylase; GES: geraniol synthase; GPP: geranyl diphosphate; GT: glucosyltransferase; HCT: hydroxycinnamoyl-CoA shikimate/quinate hydroxycinnamoyltransferase; HDR: 4-hydroxy-3-methylbut-2-enyl diphosphate reductase; HDS: 4-hydroxy-3-methylbut-2-enyldiphosphate synthase; HMGR: 3-hydroxy-3-methylglutaryl-CoA reductase; HMGS: hydroxymethylglutaryl-CoA synthase; HQT: hydroxycinnamoyl-CoA quinate hydroxycinnamoyltransferase; IPP: isopentenyl diphosphate; IRS: iridoid synthase; LAMT: loganic acid O-methyltransferase; LS: lupeol synthase; MCT: 2-C-methyl-D-erythritol 4-phosphate cytidylyltransferase; MDS: 2-C-methyl-D-erythritol 2,4-cyclodiphosphate synthase; MK: mevalonate kinase; MVD: diphosphomevalonate decarboxylase; MVP: 5-phosphomevalonate; MVPP: mevalonate-5-pyrophosphate; P450: cytochrome P450; PAL: phenylalanine-ammonia-lyase; PMK: phosphomevalonate kinase; SE: squalene epoxidase; SGD: strictosidine β-D-glucosidase; SLS: secologanin synthase; SS: squalene synthase; ST02C: terpene synthase; STR: strictosidine synthase; TDC: tryptophan decarboxylase

## 6. Transgenic Plants as Green Biofactories for the Production of Recombinant Proteins Grown in Bioreactors

Therapeutic recombinant proteins are exogenous proteins expressed in a producing organism and used for the treatment or prevention of various diseases in humans or animals [146,147,148]. Unlike artificially synthesized drugs, recombinant proteins are generally very large, complex molecules with specific mechanisms of action. Their size and complexity make the chemical synthesis of proteins very difficult, so these new drugs must be biologically produced using the protein synthesis machinery found in cells [146,149]. More than 300 protein-based drugs have been approved in the US and Europe, with proteins accounting for almost a third of all drugs under development. Almost half of the market is made up of therapeutic proteins (e.g., enzymes, antibodies, vaccines, growth factors, and cytokines), followed by industrial proteins (e.g., technical enzymes) and reagents (e.g., antibodies for protein detection and purification) [150,151,152]. Advances in recombinant protein production technologies, including engineering of expression hosts, optimization of upstream culture (e.g., bioreactor design and nutritional and physical parameters), and the development of more efficient protein extraction and purification methods are important market developments [19,153].

Production using a plant expression system is both profitable and scalable, and an interesting alternative for the pharmaceutical industry; in addition, therapeutic protein production in plants has proven to be an attractive alternative to other expression systems, such as transgenic animals, cultures of mammalian cells, yeasts, and bacteria [47,154]. Plants have provided humans with useful bioactive compounds for many centuries, but it has only been in the last few decades that it has become possible to use plants to produce specific recombinant pharmaceutical proteins. Plant production systems are extremely attractive due to the lack of risk of contamination of the final product with human or animal pathogens (e.g., bacteria, viruses, and prions) as well as bacterial toxins. One of the first pharmaceutically important proteins produced in plants was human growth hormone, which was expressed in transgenic tobacco in 1986 [42,43]. However, the structural authenticity of plant-derived recombinant proteins was confirmed in 1992, when plants were first used to produce an experimental vaccine—hepatitis B virus (HBV) surface antigen [149]. Recombinant proteins can be functionally expressed in a variety of plant systems; however, it is imperative to identify a platform that offers the best conditions for the expression and recovery of a particular protein [146,153].

**Figure 4:**
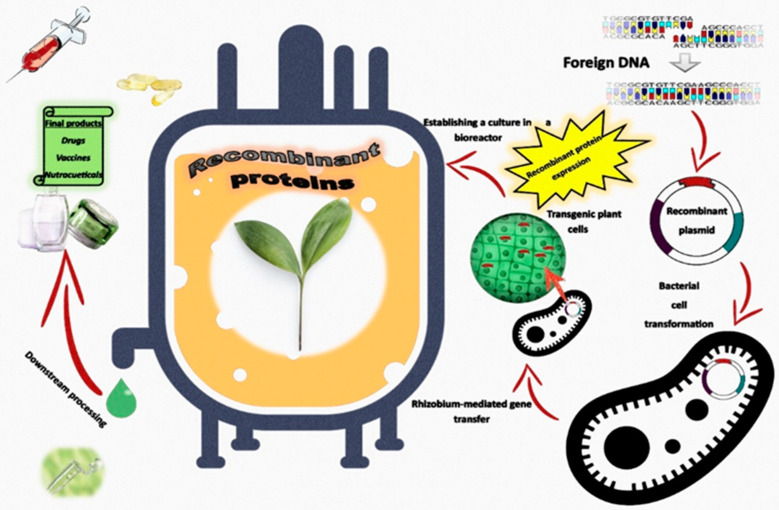
General strategy for the use of in vitro plant cultures in bioreactors for the production of recombinant proteins on an industrial scale.

There are essentially three strategies for producing recombinant proteins in plant systems: first, the use of cell culture-based systems that are equivalent to mammalian, microbial, and insect cell models; second, the transient expression of foreign genes in plant tissues, which are transformed by agro-injection or viral infection; and thirdly, the development of transgenic plants carrying stably integrated transgenes. The presented systems enable for high production efficiency through simple manipulation, thus enabling quick validation of the expression constructs and production of large amounts of recombinant protein in a short time [155,156]. The general strategy of obtaining transgenic plants, cells, or tissue cultures expressing recombinant proteins in bioreactors is shown in Figure 4.

Most research involving the production of recombinant proteins in plants has focused on early-stage goals, such as expression verification, optimization of production, and purification to some extent, and completion of initial functional tests without any cultivating trials on an industrial scale [152,157,158,159]. For example, Lim et al. examined the expression and glycosylation pattern of a recombinant therapeutic protein—GA733-FcK in transgenic *N. tabacum* seedlings [160]. Kang et al. showed high expression levels of a prostate cancer antigen with fusion of the Fc fragment of human IgG1 to the glycoprotein GA733 (PAP-IgA Fc and PAP-IgA FcK) and a high level of dimerized proteins in *N. tabacum* leaves [161]. In turn, Lu et al. revealed the expression of the colorectal cancer vaccine candidate GA733 and the antigen–antibody complex protein GA733-Fc in tobacco plant expression systems. Additionally, fusion of the Fc fragment of human IgG to the C-terminus of GA733 and the ER retention KDEL in GA733-FcK-generating oligomannose glycosylated proteins can be an ideal strategy to easily purify the recombinant GA733 vaccine candidate proteins and to enhance accumulation of the recombinant proteins with oligomannose for comparable immunogenicity of the non-KDEL-tagged mammalian-derived proteins in a plant expression system [162]. Jez et al. noted that there were expression levels up to 85 mg recombinant human erythropoietin (rhEPO)/kg in fresh leaves of *Nicotiana benthamiana (N. benthamiana*) [163]. In another example, Thomas and Walmsley showed that the transient expression of human epidermal growth factor (hEGF) in *N. benthamiana* is capable of producing large amounts of recombinant protein in combination with a P19 silencing inhibitor and codon optimized constructs [164]. Finally, Luchakivskaya et al. reported a higher activity of human interferon alpha-2b in young leaves of *Daucus carrota* plants (up to 50.7 × 10^3^ IU/g FW) compared to mature leaves, probably due to the susceptibility of this protein to degradation. Additionally, they reported that the taproot expression system could also provide sufficient protein (up to 16.5 × 10^3^ IU/g FW) and can optionally be used to produce interferon alpha-2b protein for the prevention and treatment of infectious diseases [165].

Additionally, recombinant protein production may be modulated via the CRISPR/Cas9 system. Two proteins named β(1,2)-xylose and α(1,3)-fucose were reduced in *N. tabacum* BY-2 suspension cells, knocking-out β(1,2)-xylosyltransferase and α(1,3)-fucosyltransferase, two genes that are responsible for the addition of plant-specific glycans and result in glycoproteins without plant-specific glycans [166]. The *N. benthamiana* dicer-like protein 2 and 4 double-knockout genes might produce higher amounts of human fibroblast growth factor 1 [167].

Table 2 presents selected examples of proteins obtained from transgenic plants that were cultured in bioreactors, including recombinant human butyrylcholinesterase (BChE), human cytotoxic T-lymphocyte antigen 4-immunoglobulin (hCTLA4Ig), human granulocyte-macrophage colony-stimulating factor (hGM-CSF), mouse granulo-cyte-macrophage colony stimulating factor (mGM-CSF), recombinant human serum albumin (rHSA) from *Oryza sativa*; isoform 1 of the human growth hormone (hGH1) from *Brassica oleracea*; protein A (OspA) from Borrelia burgdorferi, human monoclonal anti-body M12—a vaccine antigen—fragment C of tetanus toxin (TetC)/green fluorescent protein (GFP+), green fluorescent protein-hydrophobin fusion (GFP-HFBI) from *N. tabacum*; recombinant protein containing a fusion of rabies glycoprotein and ricin toxin B chain (rgp–rtxB) from *Solanum lycopersicum*; factor H (FH) and FH-related proteins (FHRs) from *Physcomitrella patens*; and human tissue-plasminogen activator (t-PA) protein from *Cucumis melo*. The details about the vectors or genetic construct elements, as well as the culture’s conditions in bioreactors, are also included. The presented examples showed that plant systems are good models for the production of recombinant proteins.

A common protein produced by transgenic plants is butyrylcholinesterase (BuChE), an enzyme in the serine hydrolase family that catalyzes the hydrolysis reaction of both choline and non-choline esters, including acetylcholine. Acetylcholine is an important neurotransmitter released by cholinergic neurons. Moreover, BuChE enhances the activity of proteases, including trypsin. In the human brain, its expression takes place in substantial populations of neurons and might be related to their growth during the development of the nervous system. Their enzymatic properties are altered in Alzheimer’s disease [187]. Besides somatic growth, growth hormone also has an effect on brain function. The receptors are present in areas underlying cognitive function. Growth hormone treatment can stimulate growth and improve cognition in deficient children [188,189].

There are also a group of proteins related to immune system action that could be produced by plant systems. For example, T lymphocyte antigen-4 (CTLA-4) plays an important role in the initial phase of the immune response via inhibition of T-cell effector function. *Ctla4* knockout mice exhibit lymphocyte infiltration into various organs and early lethality. Therefore, human monoclonal antibodies that target CTLA-4 increase T cell function as well as antitumor responses in cancer patients [190,191]. Granulocyte-macrophage colony-stimulating factor (GM-CSF) also plays an important immune modulatory role by enhancing the function of circulating neutrophils, monocytes, and lymphocytes during host defense as well as crucial hematopoietic growth factor. Neutrophils are also activated by a protein named protein A (OspA) expressed by *Borrelia burgdorferi* [192,193].

The crucial part of the human immune system is the complement system, which is negatively regulated by *inter alia* factor H (FH). FH was produced for the first time by *Physcomitrella patens* in a moss bioreactor. Improper FH activity results in severe kidney and eye diseases [185]. Transgenic plants are also able to produce therapeutic antibodies, which are important for treating various autoimmune, infectious, and metabolic diseases, as well as many types of cancers [147]. Monoclonal antibodies conjugated with blocked ricin, also produced by plants, create a potent immunotoxin and their role in cancer treatment is under investigation [194]. In addition, tetanus neurotoxin, also related with plant production, can possess therapeutic properties by preventing abnormal muscular concentrations [195].

Another example of plant-derived proteins are albumins, which are important carrier proteins for various substances produced in the liver. They are believed to maintain normal capillary permeability by modulating inflammation and preventing oxidative damage. The usage of albumin has been documented in various clinical situations [196,197]. Another protein that can be produced by plants is tissue plasminogen activator, a protein that converts plasminogen to plasmin—which is then followed by blood clot lysis—providing a valuable treatment for stroke patients [198]. As well, hydrophobins, which is another protein produced by planets, may have a role in medical applications such as drug delivery by creating hydrophobic surfaces [199].

## 7. Recent Patents Relating to Bioreactors for the Culture of Plant Cultures

Recent research has provided many new strategies for increasing the synthesis and/or accumulation of attractive and valuable secondary metabolites. In addition, the optimization of gene transfer into plant cells has facilitated the production of genetically modified cells, organs, or organisms that synthesize completely new recombinant proteins with a wide range of applications. The creation of strategies for increasing the productivity of plant cultures in vitro have led to the design of highly efficient systems that produce valuable secondary metabolites or recombinant proteins. A satisfactory effect is most often obtained using a combination of several different techniques and treatments, such as transgenesis, elicitation, or large-scale culture. The growth of knowledge regarding cellular processes and factors regulating biosynthetic pathways has allowed for the design of highly efficient plant in vitro cultures, the products of which can be used on an industrial scale. Recently, there has been a significant increase in the number of studies and patents related to the culture method and the use of biologically active compounds of plant origin. A good example is PhytoCellTec ™ by Mibelle Biochemistry, which consists of plant stem cells derived from callus tissue and are used as a starting point for the culture of stem cells in large-scale bioreactors. In 2008, the company registered the first *Malus domestica* preparation containing stem cell extract derived from a rare variety of Swiss apple tree (Patent number: EP1985280A2). These products are used as additives to cosmetics with anti-aging and anti-cellulite properties and are believed to protect against UV-A and UV-B radiation. In addition to the properties of various plant cells (Alp rose, *Vitis vinifera*, *Symphytum,* or Goji), the company has recently examined the potential of moss culture. Another example can be seen in the products of Innova BM. These use a range of cell suspension bioreactor cultures, including *Rosa damascena, Haberlea rhodopensis,* and *Calendula officinalis*. Their properties are used to protect epidermal stem cells against internal and external stress factors and delay the aging process. Increasing numbers of cosmetic and pharmaceutical companies have turned to plant compounds, seeing them as satisfying the demand for modern pharmaceutical and cosmetic products with exceptional properties.

In addition, an anti-aging or antioxidant composition including compounds derived from a ginseng cambium derived plant stem cell line as an active ingredient has been patented by Unhwa Corp. (Patent number:US9095532B2). The product is designed to minimize the side effects associated with existing anti-aging agents and antioxidants, making it safe for the skin. It also has antioxidant properties, inhibiting reactive oxygen species caused by exposure to UV radiation, which is the main cause of skin aging. Another example is TEUPOL 10P or 50P, which is an extract from *Ajuga reptans* cell suspension cultures introduced by ABResearch srl. The active ingredient is teupoliside (standardized at 10% or 50%) and is used in testosterone-related disorders. The same manufacturer also introduced Echinan 4P extract of *Echinacea angustifolia,* which has a wide range of neuroprotective, anti-aging, and immunomodulatory effects [11,124,200,201,202].

In addition to the many patented ingredients in cosmetics, dietary supplements, and drugs, intensive work is also underway on the technical solutions that allow for the simple and cheap culture of plant cells on a larger scale. Table 3 below shows some examples of patented bioreactors in which such cultures can be carried out.

## 8. Conclusions

The intensive development of biotechnological techniques, and thus also of transgenic plants, has had a huge impact on human life and will continue to do so. Transgenic plants offer a new approach to the production of not only specific secondary metabolites through manipulation in biosynthetic pathways, but also recombinant proteins through expression systems that greatly improve protein yield. Further increases in the amount of the desired compounds/proteins on the industrial scale have been facilitated by specially-designed bioreactors. To improve the efficiency and profitability of production, there is a great need to focus on the potential application character (patents), which complements the developed system focused on the production of specific compounds/proteins in strictly defined culture conditions. The novelty of this study is in the collection of research showing the use of transgenic cultures as small biofactories to obtain secondary metabolites and recombinant proteins in bioreactors in the biomedical industry. However, further research is needed to improve plant systems aimed at overproducing certain metabolites or proteins on an industrial scale.

## 9. Future Prospects

Intensive research related to increasing the productivity of plant cultures in vitro, despite the extensive knowledge and biotechnological tools, does not always allow to achieve satisfactory efficiency in the production of secondary metabolites or recombinant proteins. The key factors that still remain are a comprehensive understanding of metabolic pathways or an attempt to introduce complete metabolic pathways into transgenic plants cells. A promising tool with great potential for interference in the plant genome is the CRISPR/Cas9 system [203,204], which can enable manipulation that leads to the increased productivity of plant systems, especially in combination with cultivation in optimal conditions—even on a large scale—and the use of elicitation with various factors. In addition, intensive technical progress, consisting of increasing the computational capacity of microprocessors supervising the work of fully automated breeding systems, as well as the progressive miniaturization and reduction of power consumption thanks to the use of nanotechnology, will allow, in the near future, for the further improvement of the processes and increase their profitability and environmental friendliness. The new technical solutions will probably be based on natural cell–cell interactions in order to maximize the imitation of those occurring in living tissues, which is ensured, for example, by a microfluidic bioreactor [79]. Moreover, the constantly increasing number of products, such as dietary supplements or cosmetics containing components derived from plant in vitro cultures, will, in the future, further limit the exploitation of natural resources, allowing the preservation of biodiversity while increasing the supply of the desired phytochemicals.

## Figures and Tables

**Table 1:** Examples of increasing the production of selected secondary metabolites by interfering with metabolic pathways in transgenic cultures grown in bioreactors.

Plant Species/Family	Type of Culture	Vector/Genetic Construct	Type of Metabolites	Bioreactor	Medium	Effect/Yield	Ref.
*Nicotiana tabacum* L./Solanaceae	suspension culture	*Panax ginseng* dammarenediol-II synthase (PgDDS) and Cytochrome P450 716A47 (CYP716A47) under the control of the CaMV35 promoter	triterpenoid saponins (Protopanaxadiol (PPD), Dammarenediol-II)	5 L balloon-type bioreactor 2 L of Murashige & Skoog (MS) medium (working volume)	2 L of MS medium (working volume)	enhanced production of Dammarenediol-II (166.92 µg/g dry weight (DW), 1.6 mg/L) Protopanaxadiol (980.85l µg/g DW, 9.4 mg/L)	[128]
*Leonurus sibiricus* L./Lamiaceae	roots	anthocyanin pigment 1 (AtPAP1) transcription factor from Arabidopsis thaliana/pCAMBIA1305.1-AtPAP1 vector	phenolic acids	5 L sprinkle bioreactor	2.5 L Schenk & Hilde-brandt (SH) medium with 3% (*w*/*v*) sucrose	the greatest increase in DW (20.83 g/L) and highest yields of phenolic acids (chlorogenic acid 448 mg/L and caffeic acids 302 mg/L)	[129]
*Senna obtusifolia* (L.) H.S.Irwin & Barneby/Fabaceae	roots	*Panax ginseng* squalene synthase 1 gene (*PgSS1)*/pGFPGUSPlus-PgSS1 vector	pentacyclic triterpene (betulinic acid)	10 L sprinkle bioreactor	2 L of MS liquid media with 3% (*w*/*v*) sucrose	-an increase in the content of betulinic acid (38.125 mg/g DW), compared to the SOA41 hairy root line (4.213 mg/g DW)	[130]
*Nicotiana tabacum* L. cv. Petit Havana SR1/Solanaceae	hairy roots	a plastid targeted geraniol synthase gene originally isolated from *Valeriana officinalis* L. (VoGES)/pBIN2.4VoGES1 vector under the control of 35S promoter	terpenoid indole alkaloid (geraniol)	20-L wave-mixed bioreactor	2 L modified Gamborg’s B5 liquid medium	-scale production batch was successfully completed, yielding milligram quantities of geraniol.	[131]
*Centauries maritimum* (L.) Fitch/Gentianaceae	hairy roots	plasmid with GUS construct integrated into TL region of pRiA4 plasmid/GUS construct contains uidA sequence under the 70S promoter (enhancer-doubled 35S CaMV promoter), followed by NOS polyadenilation sequence.	secoiridoid glycosides (swertiamarin (SM), gentiopicrin (GP), and sweroside (SW))	RITA^®^ temporary immersion bioreactors (TIBs)	200 of liquid MS medium	-about 2–4 times higher biomass production rate and up to 8 times higher total secoiridoid glycosides production.	[132]
*Curcumae zedoariae* L./Zingiberaceae. (Christm.) Roscoe (Rhizoma)	cell suspensions	3-hydroxy-3-methylglutaryl–coenzyme A reductase (HMGR), Farnesyl-diphosphate synthase (FDS), Germacrene A synthetase GAS as well as terpene synthase (ST02C) driven by CaMV35S promoter, were separately introduced into the Agrobacterium GV3101	sesquiterpenes (β-elemene)	2 L stir-tank and airlift bioreactor	liquid MS medium containing 28 g L^−1^ sucrose, 0.5 mg L^−1^ 6-BA, 1.0 mg L^−1^ naphthylacetic acid (NAA), 1.0 mg L^−1^ 2,4-D	-highest β-elemene content of 0.22% (*w*/*v*) was detected in ST02C-transformed lines	[133]
*Vinca minor* L./Apocynaceae	cell suspensions	Tryptophan decarboxylase (TDC) and strictosidine synthase (STR) genes	monomeric eburnamine-type indole alkaloid vincamine	5-L stirred tank bioreactor	MS medium with 2% sucrose	-only PVG3 line registered a twofold increase in total alkaloid content (2.1 ± 0.1% DW) and showed vincamine presence (0.003 ± 0.001% DW) which was further enhanced at the bioreactor level (2.7 ± 0.3 and 0.005 ± 0.001% DW, respectively)	[134]

**Table 2:** Overproduction of recombinant proteins in transgenic cultures grown in bioreactors.

Name of the Species/Family	Type of Culture	Vector/Genetic Construct Elements	Recombinant Protein	Type of Bioreactor	Medium/ Elicitors Used	Effect/Yield	Ref.
*Oryza sativa* L./ Poaceae	suspension culture	metabolically-regulated promoter, rice alpha-amylase 3D (*RAmy3D*)	recombinant human butyrylcholinesterase (BChE),	5-L stirred-tank bioreactor	-half-strength sucrose of the culture medium	-the method significantly improved the maximum accumulation level, purity, and productivity of the recombinant protein	[168]
*Oryza sativa* L./ Poaceae	suspension culture	metabolically-regulated promoter, rice alpha-amylase 3D (*RAmy3D*)	recombinant human butyrylcholinesterase (BChE),	40-L stainless-steel stirred tank bioreactors (STB) bioreactor	-NB + S medium contains 30 g sucrose/L, while NB + 0.5xS contains 15 g sucrose/L	-maximum total active rrBChE production level of 46–58 µg/g fresh weight (FW) in four cycles over 82 days -overall volumetric oxygen mass transfer coefficient (k_L_a) in the pilot-scale STB to be equivalent to the lab-scale STB volumetric productivity to 85 µg/g FW and 387 µg/L/day	[169]
*Oryza sativa* L./ Poaceae	suspension culture	α-amylase 3D (RAmy3D) promoter	*N*-glycosylation of recombinant human butyrylcholinesterase (BChE)	5 L bioreactor	-using normal sugar-free (NB-S) media with no kifunensine treatment	-total active rrBChE production level of 79 ± 2 µg/g FW or 7.5 ± 0.4 mg/L in the presence of kifunensine	[170]
*Oryza sativa* L./ Poaceae	suspension culture	alpha amylase 3D (RAmy3D)	tetrameric form of recombinant butyrylcholinesterase (BChE	5 L bioreactor	-fresh liquid NB + S medium	-maximum yield of 1.6 mg BChE/L of culture during the second expression phase	[171]
*Oryza sativa* L./ Poaceae	suspension culture	RAmy3D promoter	human cytotoxic T-lymphocyte antigen 4-immunoglobulin (hCTLA4Ig)	3-L multi-bioreactor	-AA medium (1.4 L), except the volume of amino acid solution	-total protein concentration was at levels from 301.0 to 782.8 mg/L	[172]
*Oryza sativa* L./ Poaceae	suspension culture	RAmy3D promoter	human cytotoxic T-lymphocyte antigen 4-immunoglobulin (hCTLA4Ig)	stirred-tank reactors (5-L STR)	-AA medium (2.1 L) except 10% (*v*/*v*) amino acid mixture	-the results in both disposable bioreactors presented similar values of the maximum cell density (11.9 g DCW/L and 12.6 g DCW/L), the doubling time (4.8 and 5.0 days) and the maximum hCTLA4Ig concentration (43.7 and 43.3 mg/L).	[173]
*Oryza sativa* L./ Poaceae	suspension culture	RAmy3D promoter	human cytotoxic T-lymphocyte antigen 4-immunoglobulin (hCTLA4Ig)	7-L bioreactor, 15-L stirred-tank bioreactor	AA medium (2.3 L)	-maximum hCTLA4Ig level was 76.5 mg/L at day 10	[174]
*Oryza sativa* L./ Poaceae	suspension culture	RAmy3D promoter	human granulocyte-macrophage colony-stimulating factor (hGM-CSF)	2-L bioreactor, 5-L stirred-tank bioreactor	-N6 medium 0.2 mg/L kinetin, 2 mg/L, 2mg/L 2,4-D, 30 g/L sucrose	-induction using sugar free media produced 33% more hGM-CSF -using buffer exchange when CM-Sepharose was used as a cationic exchange resin, optimal pH for binding was 4.8 and adsorption yield was 77%. -DEAE-Sepharose was used as an anionic exchange resin, it was 5.5 (74%). -without buffer exchange, optimal pH was 4.6 (84%).	[175]
*Brassica oleracea var. italica (broccoli)*/ Brassicaceae	hairy roots	pCAMBIA1105.1 binary vector	isoform 1 of the human growth hormone (hGH1)	1.5-L mesh airlift bioreactor	-1.25 L of Schenk & Hildebrandt (SH) medium supplemented with sucrose 30 g/L and (NH4)2SO4 300 mg/L.	-the production of hGH1 was 5.1 ± 0.42 µg/g dry weight (DW) for flask cultures and 7.8 ± 0.3 µg/g DW for the bioreactor, with a capacity of 0.68 ± 0.05 and 1, 5 ± 0.06 µg/g DW days	[176]
*Nicotiana tabacumcv Petit Havana*/ Solanaceae	suspension culture/leaves	pOA:YFP4411	protein A (OspA) from Borrelia burgdorferi	immersion bioreactors (TIBs) using AlkaBurst^TM^	-1-L and 0.3-L Murashige & Skoog (MS) media	-OspA expression up to 7.6% TSP with a maximum OspA yield of about 108 mg	[177]
*Oryza sativa* L./ Poaceae	suspension culture	a-amylase gene aAmy8 promotor/Gateway-compatible binary T-DNA destination vector	mouse granulocyte-macrophage colony stimulating factor (mGM-CSF)	2-L bioreactor	-1.5 L of N6 medium	-the highest yield of rmGM-CSF was 24.6 mg/L	[178]
*Nicotiana tabacum* L./ Solanaceae	suspension culture BY-2	binary vector pTRAkc-MTAD	human monoclonal antibody M12	200-L Orbitally-Shaken Disposable Bioreactor, 20-L Nalgene polycarbonate carboy vessels	-MSN medium	-final cell fresh weights of 300–387 g/L and M12 yields of 20 mg/L -resulting in an overall M12 recovery of 75–85% and a purity of >95%	[179]
*Oryza sativa* L./ Poaceae	suspension culture	RAmy3D promoter	recombinant human butyrylcholinesterase (BChE)	5-L stirred-tank bioreactor	-3 L of NB + Smedium	-maximum total active rrBChE (77 μg/g FW) and 1.6-fold increase of total active rrBChE specific productivity (86 μg/g DW/day) compared to the two-stage batch cultures.	[180]
*Solanum lycopersicum* L./ Solanaceae	hairy roots	CaMV35S promoter	recombinant protein containing a fusion of rabies glycoprotein and ricin toxin B chain (rgp–rtxB)	5 L bioreactor Bench-top fermenter (Bioflo-3000)	-the quantity of 2.5 L of 1/2 MS medium with B5 vitamins and 3% sucrose	-biomass yield 197.4 (g/L) -RGP RTB 7.84 (µg/g) -the efficiency of the bioreactor in terms of protein expression remained relatively lower than that of the shake flask, which may be due to callogenesis of the root tissues in the bioreactor.	[181]
*Nicotiana tabacum* L./ Solanaceae	suspension cultures (and calli)/leaves	Plasmid pFMGFP	a vaccine antigen, fragment C of tetanus toxin (TetC)/green fluorescent protein (GFP+)	2 L bioreactor	MS medium supplemented with 0.1 lM TDZ	-GFP+ yield reached 660 mg/L of bioreactor (33% TSP), and TetC accumulated to about 95 mg/L (8% TSP)	[182]
*Oryza sativa* L./ Poaceae	suspension culture	α-amylase gene promoter, *RAmy3D*p/*αAmy3*p	recombinant human serum albumin (rHSA)	2-L airlift and a 2-L stirred tank bioreactor	MS medium	-rHSA production has been enriched to 45 mg/L in plant culture	[183]
*Nicotina tabacum* L./ Solanaceae	suspension cell cultures BY-2	vector pCaMterX enhanced virus 35S promoter	green fluorescent protein-hydrophobin fusion (GFP-HFBI)	30-L bioreactor, 600-L standard stirred tank bioreactor	MS-medium	-HFB-fusion technology in large-scale tobacco BY-2 suspension cell culture, formation of protein bodies and efficient purification of GFP-HFBI fusion by aqueous two-phase separation (ATPS) -GFP-HFBI titer reached a level of 0.30 ± 0.018 g/L, corresponding to 16.5% of TSP (total soluble protein)	[184]
*Physcomitrella patens* (Hedw.) Bruch & Schimp/ Funariaceae	whole plant	MFHR1 construct	factor H (FH) and FH-related proteins (FHRs)	5-L bioreactor	-fresh medium with the addition of 5 μM naphthaleneacetic acid (NAA)	-it was obtained 17 mg of MFHR1 protein	[185]
*Cucumis melo* L./ Cucurbitaceae	hairy roots	binary plasmid p221 that included cauliflower mosaic virus 35S promoter, tobacco etch virus (TEV) leader sequence and 35S terminator	human tissue-plasminogen activator (t-PA) protein	18 L bioreactor	-MS, Woody Plant Me-dium (WPM), B5 medium	-biomass accumulation 615.4 g/FW in MS medium, 457.6 g/FW in B5 medium and 621.8 g/FW in WPM medium -the maximum content of t-PA 0.46 μg/mg TSP was obtained in the cultures grown on the B5 medium, and then the content of t-PA 0.33 and 0.40 μg/mg TSP in the cultures grown on the MS and WPM medium	[186]

**Table 3:** Selected examples of patents relating to bioreactors for the culture of plant material.

Patent/Patent Application N^o^	Assignee	Type of Plant Culture	Year
WO2012044239A1	-	Tissue cultures	2012
CN102408991B	Bright Oceans Corp. (Shaanxi, China)	Cells, tissues and organs	2014
CN103120126B	Nanjing University Nanjing University of Science and Technology	Plant tissue culture	2014
US20140026260A1	Worcester Polytechnic Institute	Tissues and organs, whole plants	2014
WO2015066779A1	-	Tissue culture	2015
EP2674479B1	Eppendorf AG (Hamburg, Germany)	Cell culture	2015
CN103270946B	Nanjing Biofunction Biological Science & Technology Co Ltd. (Nanjing, China)	Plant tissue culture	2016
WO2016092098A1	-	Cell or tissue cultures	2016
CN104770304B	Nanjing Biofunction Biological Science & Technology Co Ltd.	Plant tissue culture	2017
EP3069591B1	Fibria Celulose SA (Sao Paulo, Brazil)	Plant tissue culture	2018
CN104379722B	Eppendorf AG (Hamburg, Germany)	Cell culture	2018
USD822223S1	University of Guelph	Tissue culture	2018
EP3502229A1	Evologic Technologies GmbH (Vienna, AT)	Hairy root cultures	2019
US20190282983A1	Life Technologies Corp (Carlsbad, CA)	Cell culture	2019
CN208857314U	PURUIKANG BIOTECHNOLOGY CO Ltd. (Shenzhen, China)	plant cell, organ	2019
CN111226794A	Beijing Forestry University	Mature somatic embryos	2020
CN212247083U	Zhejiang University of Technology ZJUT	Cell culture	2020
ES2763637B2	Institut Recerca i Tecnologia Agroalimentaries IRTA	Plant tissues, organs, seeds and/or plant cells	2020
US20200032185A1	Oklahoma State University	Cell culture	2020
US20200230568A1	ABEC Inc.	Cell culture	2020
US20200339931A1	Sartorius Stedim Biotech GmbH (Goettingen Germany)	Cell culture	2020
US20210130765A1	Ori biotech Ltd. (London, United Kingdom)	Cell culture	2021
US20210214668A1	Membio Inc (Mississauga, ON, Canada)	Cell or tissue culture	2021

## Data Availability

Not applicable.

## References

[1] Calvet-Mir L., Salpeteur M. (2016). Humans, plants, and networks: A critical review. Environ. Soc. Adv. Res..

[2] DelSesto M. (2020). People–plant interactions and the ecological self. Plants People Planet..

[3] Henkhaus N., Bartlett M., Gang D., Grumet R., Jordon-Thaden I., Lorence A., Lyons E., Miller S., Murray S., Nelson A. (2020). Plant science decadal vision 2020–2030: Reimagining the potential of plants for a healthy and sustainable future. Plant Direct..

[4] Salam S.A., Javed M.S., Toor M.D., Adnan M., Awais M., Din M.M.U., Saeed M.S., ur Rehman F., Tampubolon K. (2020). Influence of Industrial Waste Water on Soil and Plants: A Review. Curr. Res. Agric. Farming..

[5] Altemimi A., Lakhssassi N., Baharlouei A., Watson D.G., Lightfoot D.A. (2017). Phytochemicals: Extraction, isolation, and identification of bioactive compounds from plant extracts. Plants.

[6] Guerriero G., Berni R., Muñoz-Sanchez J.A., Apone F., Abdel-Salam E.M., Qahtan A.A., Alatar A.A., Cantini C., Cai G., Hausman J.F. (2018). Production of plant secondary metabolites: Examples, tips and suggestions for biotechnologists. Genes.

[7] Manousi N., Sarakatsianos I., Samanidou V. (2019). Extraction Techniques of Phenolic Compounds and Other Bioactive Compounds From Medicinal and Aromatic Plants. Eng. Tools Beverage Ind..

[8] Li Y., Kong D., Fu Y., Sussman M.R., Wu H. (2020). The effect of developmental and environmental factors on secondary metabolites in medicinal plants. Plant Physiol. Biochem..

[9] Yang L., Wen K.S., Ruan X., Zhao Y.X., Wei F., Wang Q. (2018). Response of plant secondary metabolites to environmental factors. Molecules.

[10] Espinosa-Lea C.A., Puente-Garza C.A., García-Lara S. (2018). In vitro plant tissue culture: Means for production of biological active compounds. Planta.

[11] Chandran H., Meena M., Barupal T., Sharma K. (2020). Plant tissue culture as a perpetual source for production of industrially important bioactive compounds. Biotechnol. Rep. (Amst).

[12] Matsuura H.N., Malik S., de Costa F., Yousefzadi M., Mirjalili M.H., Arroo R., Bhambra A.S., Strnad M., Bonfill M., Fett-Neto A.G. (2018). Specialized Plant Metabolism Characteristics and Impact on Target Molecule Biotechnological Production. Mol. Biotechnol..

[13] Ncube B., Van Staden J. (2015). Tilting plant metabolism for improved metabolite biosynthesis and enhanced human benefit. Molecules.

[14] Georgiev M.I., Agostini E., Ludwig-Müller J., Xu J. (2012). Genetically transformed roots: From plant disease to biotechnological resource. Trends Biotechnol..

[15] Roy A. (2020). Hairy Root Culture an Alternative for Bioactive Compound Production from Medicinal Plants. Curr. Pharm. Biotechnol..

[16] Gutierrez-Valdes N., Häkkinen S.T., Lemasson C., Guillet M., Oksman-Caldentey K.M., Ritala A., Cardon F. (2020). Hairy Root Cultures—A Versatile Tool With Multiple Applications. Front. Plant Sci..

[17] Shi M., Liao P., Nile S.H., Georgiev M.I., Kai G. (2021). Biotechnological Exploration of Transformed Root Culture for Value-Added Products. Trends Biotechnol..

[18] Merlin M., Gecchele E., Capaldi S., Pezzotti M., Avesani L. (2014). Comparative evaluation of recombinant protein production in different biofactories: The green perspective. Biomed Res. Int..

[19] Tripathi N.K., Shrivastava A. (2019). Recent Developments in Bioprocessing of Recombinant Proteins: Expression Hosts and Process Development. Front. Bioeng. Biotechnol..

[20] Alireza T., Nader R.E., El-Shemy H. (2015). Molecular Farming in Plants. Plants for the Future.

[21] Georgiev M.I., Eibl R., Zhong J.J. (2013). Hosting the plant cells in vitro: Recent trends in bioreactors. Appl. Microbiol. Biotechnol..

[22] Valdiani A., Hansen O.K., Nielsen U.B., Johannsen V.K., Shariat M., Georgiev M.I., Omidvar V., Ebrahimi M., Tavakoli Dinanai E., Abiri R. (2019). Bioreactor-based advances in plant tissue and cell culture: Challenges and prospects. Crit. Rev. Biotechnol..

[23] Mamun N.H.A., Egertsdotter U., Aidun C.K. (2015). Bioreactor technology for clonal propagation of plants and metabolite production. Front. Biol..

[24] Xu J., Zhang N. (2014). On the way to commercializing plant cell culture platform for biopharmaceuticals: Present status and prospect. Pharm. Bioprocess..

[25] Moon K.B., Park J.S., Park Y., Song I.J., Lee H.J., Cho H.S., Jeon J.H., Kim H.S. (2020). Development of systems for the production of plant-derived biopharmaceuticals. Plants.

[26] Page M.J., McKenzie J.E., Bossuyt P.M., Boutron I., Hoffmann T.C., Mulrow C.D., Shamseer L., Tetzlaff J.M., Akl E.A., Brennan S.E. (2021). The PRISMA 2020 statement: An updated guideline for reporting systematic reviews. BMJ.

[27] Maghari B.M., Ardekani A.M. (2011). Genetically modified foods and social concerns. Avicenna J. Med. Biotechnol..

[28] Oliver M.J. (2014). Why we need GMO crops in agriculture. Mo. Med..

[29] Cano A., Morgado C. (2017). The role of biotechnology in agricultural production and food supply. Cienc. Investig. Agrar..

[30] Tutelyan V.A. (2013). Chapter 2—World Production of Genetically Engineered Crops. Genetically Modified Food Sources.

[31] ISAAA (2019). ISAAA Brief 55-2019: Executive Summary Biotech Crops Drive Socio-Economic Development and Sustainable Environment in the New Frontier. Int. Serv. Acquis. Agri-Biotech Appl..

[32] ISAAA Pocket K No. 16: Biotech Crop Highlights in 2019. https://www.isaaa.org/resources/publications/pocketk/16/.

[33] James C. (2017). Global Status of Commercialized Biotech/GM Crops in 2017: Biotech Crop Adoption Surges as Economic Benefits Accumulate in 22 Years. https://www.isaaa.org/resources/publications/briefs/53/download/isaaa-brief-53-2017.pdf.

[34] Fedoroff N.V. (2015). Food in a future of 10 billion. Agric. Food Secur..

[35] Raman R. (2017). The impact of Genetically Modified (GM) crops in modern agriculture: A review. GM Crop. Food..

[36] Pellegrini P.A. (2013). Anomalies in the Early Stages of Plant Transgenesis: Interests and Interpretations Surrounding the First Transgenic Plants. Hist. Ciencias Saude—Mang..

[37] Barton K.A., Binns A.N., Matzke A.J.M., Chilton M.D. (1983). Regeneration of intact tobacco plants containing full length copies of genetically engineered T-DNA, and transmission of T-DNA to R1 progeny. Cell.

[38] Gelvin S.B. (2003). Agrobacterium-mediated plant transformation: The biology behind the “gene-jockeying” tool. Microbiol. Mol. Biol. Rev..

[39] Lacroix B., Citovsky V. (2013). The roles of bacterial and host plant factors in Agrobacterium-mediated genetic transformation. Int. J. Dev. Biol..

[40] Lawlor D.W. (2013). Genetic engineering to improve plant performance under drought: Physiological evaluation of achievements, limitations, and possibilities. J. Exp. Bot..

[41] Hussain A., Ahmed I., Nazir H., Ullah I., Leva A., Rinaldi L. (2012). Plant Tissue Culture: Current Status and Opportunities. Recent Advances in Plant In Vitro Culture.

[42] Sharma A.K., Sharma M.K. (2009). Plants as bioreactors: Recent developments and emerging opportunities. Biotechnol. Adv..

[43] Yao J., Weng Y., Dickey A., Wang K.Y. (2015). Plants as factories for human pharmaceuticals: Applications and challenges. Int. J. Mol. Sci..

[44] Paul J.Y., Khanna H., Kleidon J., Hoang P., Geijskes J., Daniells J., Zaplin E., Rosenberg Y., James A., Mlalazi B. (2017). Golden bananas in the field: Elevated fruit pro-vitamin A from the expression of a single banana transgene. Plant Biotechnol. J..

[45] Zhang C., Wohlhueter R., Zhang H. (2016). Genetically modified foods: A critical review of their promise and problems. Food Sci. Hum. Wellness.

[46] Liao P., Chen X., Wang M., Bach T.J., Chye M.L. (2018). Improved fruit α-tocopherol, carotenoid, squalene and phytosterol contents through manipulation of *Brassica juncea* 3-HYDROXY-3-METHYLGLUTARYL-COA SYNTHASE1 in transgenic tomato. Plant Biotechnol. J..

[47] Xu J., Towler M., Weathers P.J., Pavlov A., Bley T. (2018). Platforms for Plant-Based Protein Production. Bioprocessing of Plant In Vitro Systems. Reference Series in Phytochemistry.

[48] Babich O., Sukhikh S., Pungin A., Ivanova S., Asyakina L., Prosekov A. (2020). Modern Trends in the In Vitro Production and Use of Callus, Suspension Cells and Root Cultures of Medicinal Plants. Molecules.

[49] Häkkinen S.T., Reuter L., Nuorti N., Joensuu J.J., Rischer H., Ritala A. (2018). Tobacco BY-2 media component optimization for a cost-efficient recombinant protein production. Front. Plant Sci..

[50] Kowalczyk T., Wieczfinska J., Skała E., Śliwiński T., Sitarek P. (2020). Transgenesis as a tool for the efficient production of selected secondary metabolites from in vitro plant cultures. Plants.

[51] Dong O.X., Ronald P.C. (2019). Genetic engineering for disease resistance in plants: Recent progress and future perspectives. Plant Physiol..

[52] Pickens L.B., Tang Y., Chooi Y.H. (2011). Metabolic engineering for the production of natural products. Annu. Rev. Chem. Biomol. Eng..

[53] Jan R., Asaf S., Numan M., Lubna K.M. (2021). Plant secondary metabolite biosynthesis and transcriptional regulation in response to biotic and abiotic stress conditions. Agronomy.

[54] Kamthan A., Chaudhuri A., Kamthan M., Datta A. (2016). Genetically modified (GM) crops: Milestones and new advances in crop improvement. Theor. Appl. Genet..

[55] Kumar K., Gambhir G., Dass A., Tripathi A.K., Singh A., Jha A.K., Yadava P., Choudhary M., Rakshit S. (2020). Genetically modified crops: Current status and future prospects. Planta.

[56] Bailey-Serres J., Parker J.E., Ainsworth E.A., Oldroyd G.E.D., Schroeder J.I. (2019). Genetic strategies for improving crop yields. Nature.

[57] Ahmar S., Gill R.A., Jung K.H., Faheem A., Qasim M.U., Mubeen M., Zhou W. (2020). Conventional and molecular techniques from simple breeding to speed breeding in crop plants: Recent advances and future outlook. Int. J. Mol. Sci..

[58] Blair M.W., Cortés A.J., This D. (2016). Identification of an ERECTA gene and its drought adaptation associations with wild and cultivated common bean. Plant Sci..

[59] Cortés A.J., Chavarro M.C., Madriñán S., This D., Blair M.W. (2012). Molecular ecology and selection in the drought-related Asr gene polymorphisms in wild and cultivated common bean (*Phaseolus vulgaris* L.). BMC Genet..

[60] Cortés A.J., This D., Chavarro C., Madriñán S., Blair M.W. (2012). Nucleotide diversity patterns at the drought-related DREB2 encoding genes in wild and cultivated common bean (*Phaseolus vulgaris* L.). Theor. Appl. Genet..

[61] Wheeler J.A., Cortés A.J., Sedlacek J., Karrenberg S., van Kleunen M., Wipf S., Hoch H., Bossdorf O., Rixen C. (2016). The snow and the willows: Earlier spring snowmelt reduces performance in the low-lying alpine shrub *Salix herbacea*. J. Ecol..

[62] Wheeler J.A., Hoch G., Cortés A.J., Sedlacek J., Wipf S., Rixen C. (2014). Increased spring freezing vulnerability for alpine shrubs under early snowmelt. Oecologia.

[63] Rani S.J., Usha R. (2013). Transgenic plants: Types, benefits, public concerns and future. J. Pharm. Res..

[64] Prakash D., Verma S., Bhatia R., Tiwary B.N. (2011). Risks and Precautions of Genetically Modified Organisms. Int. Sch. Res. Not..

[65] Goldstein D.A., Thomas J.A. (2004). Biopharmaceuticals derived from genetically modified plants. QJM.

[66] Apone F., Barbulova A., Colucci M.G. (2019). Plant and microalgae derived peptides are advantageously employed as bioactive compounds in cosmetics. Front. Plant Sci..

[67] Baenas N., Belović M., Ilic N., Moreno D.A., García-Viguera C. (2019). Industrial use of pepper (*Capsicum annum* L.) derived products: Technological benefits and biological advantages. Food Chem..

[68] Dias R., Oliveira H., Fernandes I., Simal-Gandara J., Perez-Gregorio R. (2021). Recent advances in extracting phenolic compounds from food and their use in disease prevention and as cosmetics. Crit. Rev. Food Sci. Nutr..

[69] Eibl R., Meier P., Stutz I., Schildberger D., Hühn T., Eibl D. (2018). Plant cell culture technology in the cosmetics and food industries: Current state and future trends. Appl. Microbiol. Biotechnol..

[70] Devi J., Kumar R., Singh K., Gehlot A., Bhushan S., Kumar S. (2021). In vitro adventitious roots: A non-disruptive technology for the production of phytoconstituents on the industrial scale. Crit. Rev. Biotechnol..

[71] Isah T., Umar S., Mujib A., Sharma M.P., Rajasekharan P.E., Zafar N., Frukh A. (2018). Secondary metabolism of pharmaceuticals in the plant in vitro cultures: Strategies, approaches, and limitations to achieving higher yield. Plant Cell Tissue Organ Cult..

[72] Jesionek A., Kokotkiewicz A., Krolicka A., Zabiegala B., Luczkiewicz M. (2018). Elicitation strategies for the improvement of essential oil content in *Rhododendron tomentosum* (*Ledum palustre*) bioreactor-grown microshoots. Ind. Crops Prod..

[73] Pandey P., Singh S., Banerjee S. (2019). *Ocimum basilicum* suspension culture as resource for bioactive triterpenoids: Yield enrichment by elicitation and bioreactor cultivation. Plant Cell. Tissue Organ Cult..

[74] Zarei A., Behdarvandi B., Tavakouli Dinani E., Maccarone J. (2021). *Cannabis sativa* L. photoautotrophic micropropagation: A powerful tool for industrial scale in vitro propagation. Vitr. Cell. Dev. Biol. Plant.

[75] Udomsin O., Yusakul G., Kitisripanya T., Juengwatanatrakul T., Putalun W. (2020). The Deoxymiroestrol and Isoflavonoid Production and Their Elicitation of Cell Suspension Cultures of *Pueraria candollei* var. *mirifica*: From Shake Flask to Bioreactor. Appl. Biochem. Biotechnol..

[76] Routien J.B., Nickell L.G. (1956). Cultivation of plant tissue. USA Pat. 2.

[77] Murashige T., Skoog F. (1962). A Revised Medium for Rapid Growth and Bio Assays with Tobacco Tissue Cultures. Physiol. Plant..

[78] Wang S.J., Zhong J.J., Yang S.T. (2007). Bioreactor Engineering. Bioprocessing for Value-Added Products from Renewable Resources.

[79] Finkbeiner T., Manz C., Raorane M.L., Metzger C., Schmidt-Speicher L., Shen N., Ahrens R., Maisch J., Nick P., Guber A.E. (2022). A modular microfluidic bioreactor to investigate plant cell–cell interactions. Protoplasma.

[80] Wang B., Wang Z., Chen T., Zhao X. (2020). Development of Novel Bioreactor Control Systems Based on Smart Sensors and Actuators. Front. Bioeng. Biotechnol..

[81] Malhotra N. (2019). Bioreactors Design, Types, Influencing Factors and Potential Application in Dentistry. A Literature Review. Curr. Stem Cell Res. Ther..

[82] Ozgun H., Dereli R.K., Ersahin M.E., Kinaci C., Spanjers H., Van Lier J.B. (2013). A review of anaerobic membrane bioreactors for municipal wastewater treatment: Integration options, limitations and expectations. Sep. Purif. Technol..

[83] Chastang T., Pozzobon V., Taidi B., Courot E., Clement C., Pereau D. (2018). Resveratrol production by grapevine cells in fed-batch bioreactor: Experiments and modelling. Biochem. Eng. J..

[84] Carolin C.F., Kumar P.S., Joshiba G.J., Kumar V.V. (2021). Analysis and removal of pharmaceutical residues from wastewater using membrane bioreactors: A review. Environ. Chem. Lett..

[85] Terrier B., Courtois D., Hénault N., Cuvier A., Bastin M., Aknin A., Dubreuil J., Pétiard V. (2007). Two new disposable bioreactors for plant cell culture: The wave and undertow bioreactor and the slug bubble bioreactor. Biotechnol. Bioeng..

[86] Eibl R., Eibl D. (2008). Design of bioreactors suitable for plant cell and tissue cultures. Phytochem. Rev..

[87] Pinto D., da Silva C.L., Cabral J. (2018). Scalable Expansion of Mesenchymal Stem/Stromal Cells in Bioreactors: A Focus on Hydrodynamic Characterization. Reference Module in Biomedical Sciences.

[88] Georgiev V., Schumann A., Pavlov A., Bley T. (2014). Temporary immersion systems in plant biotechnology. Eng. Life Sci..

[89] Yudharaj P., Shankar M., Sowjanya R., Sireesha B., Naik E.A., Priyadarshini R.J. (2016). Importance and uses of medicinal plants–An overview. Int. J. Preclin. Pharm. Res..

[90] Gaosheng H., Jingming J., Leva A., Rinaldi L. (2012). Production of Useful Secondary Metabolites Through Regulation of Biosynthetic Pathway in Cell and Tissue Suspension Culture of Medicinal Plants. Recent Advances in Plant In Vitro Culture.

[91] Tiago O., Maicon N., Ivan R.C., Diego N.F., Vinícius J.S., Mauricio F., de Alan J.P., de Velci Q.S. (2017). Plant secondary metabolites and its dynamical systems of induction in response to environmental factors: A. review. African. J. Agric. Res..

[92] Hussein R.A., El-Anssary A.A., Builders P. (2018). Plants Secondary Metabolites: The Key Drivers of the Pharmacological Actions of Medicinal Plants. Herbal Medicine.

[93] Wang S., Alseekh S., Fernie A.R., Luo J. (2019). The Structure and Function of Major Plant Metabolite Modifications. Mol. Plant..

[94] Karuppusamy S. (2009). A review on trends in production of secondary metabolites from higher plants by in vitro tissue, organ and cell cultures. J. Med. Plants Res..

[95] Verpoorte R., Contin A., Memelink J. (2002). Biotechnology for the production of plant secondary metabolites. Phytochem. Rev..

[96] Atanasov A.G., Waltenberger B., Pferschy-Wenzig E.M., Linder T., Wawrosch C., Uhrin P., Temml V., Wang L., Schwaiger S., Heiss E.H. (2015). Discovery and resupply of pharmacologically active plant-derived natural products: A review. Biotechnol. Adv..

[97] Lahlou M. (2013). The Success of Natural Products in Drug Discovery. Pharmacol. Pharm..

[98] Chitra J., Khatana S., Vijayvergia R. (2019). Bioactivity of secondary metabolites of various plants: A. review. Int. J. Pharm. Sci. Res..

[99] Namdeo A.G. (2007). Plant Cell Elicitation for Production of Secondary Metabolites: A Review. Phcog. Rev..

[100] Atanasov A.G., Zotchev S.B., Dirsch V.M., Orhan I.E., Banach M., Rollinger J.M., Barreca D., Weckwerth W., Bauer R., Bayer E.A. (2021). Natural products in drug discovery: Advances and opportunities. Nat. Rev. Drug Discov..

[101] Gonçalves S., Romano A., Vijayakumar R., Raja S. (2018). Production of Plant Secondary Metabolites by Using Biotechnological Tools. Secondary Metabolites-Sources and Applications.

[102] Isah T. (2019). Stress and defense responses in plant secondary metabolites production. Biol. Res..

[103] Hussain M.S., Fareed S., Ansari S., Rahman M.A., Ahmad I.Z., Saeed M. (2012). Current approaches toward production of secondary plant metabolites. J. Pharm. Bioallied Sci..

[104] Yue W., Ming Q.L., Lin B., Rahman K., Zheng C.J., Han T., Qin L.P. (2016). Medicinal plant cell suspension cultures: Pharmaceutical applications and high-yielding strategies for the desired secondary metabolites. Crit. Rev. Biotechnol..

[105] Lu X., Tan K., Li P. (2016). Plant metabolic engineering strategies for the production of pharmaceutical terpenoids. Front. Plant Sci..

[106] Barh D., Azevedo V. (2017). Omics Technologies and Bio-Engineering.

[107] National Academies of Sciences, Engineering, and Medicine, Division on Earth and Life Studies, Board on Agriculture and Natural Resources, Committee on Genetically Engineered Crops (2016). Past Experience and Future Prospects. Genetically Engineered Crops: Experiences and Prospects.

[108] Miralpeix B., Rischer H., Hakkinen S., Ritala A., Seppanen-Laakso T., Oksman-Caldentey K.-M., Capell T., Christou P. (2013). Metabolic Engineering of Plant Secondary Products: Which Way Forward?. Curr. Pharm. Des..

[109] Aftab T., Rehman K. (2020). Medicinal and Aromatic Plants. Expanding their Horizons through Omics.

[110] Bahramnejad B., Naji M., Bose R., Jha S. (2019). A critical review on use of *Agrobacterium rhizogenes* and their associated binary vectors for plant transformation. Biotechnol. Adv..

[111] Kang Y.M., Lee O.S., Jung H.Y., Kang S.M., Lee B.H., Karigar C., Prasad T., Bahk J.D., Choi M.S. (2005). Overexpression of hyoscyamine 6β-hydroxylase(h6h) gene and enhanced production of tropane alkaloids in *Scopolia parviflora* hairy root lines. J. Microbiol. Biotechnol..

[112] Riad M., Hithe C.C. (2021). Scopolamine. StatPearls.

[113] Kowalczyk T., Sitarek P., Toma M., Picot L., Wielanek M., Skała E., Śliwiński T. (2020). An extract of transgenic *Senna obtusifolia* L. Hairy roots with overexpression of PgSS1 gene in combination with chemotherapeutic agent induces apoptosis in the leukemia cell line. Biomolecules.

[114] Zhang X., Hu J., Chen Y. (2016). Betulinic acid and the pharmacological effects of tumor sup-pression (Review). Mol. Med. Rep..

[115] Shim J.S., Lee O.R., Kim Y.J., Lee J.H., Kim J.H., Jung D.Y., In J.G., Lee B.S., Yang D.C. (2010). Overexpression of PgSQS1 increases ginsenoside production and negatively affects ginseng growth rate in *Panax ginseng*. J. Ginseng Res..

[116] Kim J.H. (2018). Pharmacological and medical applications of *Panax ginseng* and ginseno-sides: A review for use in cardiovascular diseases. J. Ginseng Res..

[117] Jian W., Cao H., Yuan S., Liu Y., Lu J., Lu W., Li N., Wang J., Zou J., Tang N. (2019). SlMYB75, an MYB-type transcription factor, promotes anthocyanin accumulation and enhances volatile aroma production in tomato fruits. Hortic. Res..

[118] Bassolino L., Zhang Y., Schoonbeek H.J., Kiferle C., Perata P., Martin C. (2013). Accumulation of anthocyanins in tomato skin extends shelf life. New Phytol..

[119] Sitarek P., Kowalczyk T., Rijo P., Białas A.J., Wielanek M., Wysokińska H., Garcia C., Toma M., Śliwiński T., Skała E. (2018). Over-Expression of AtPAP1 Transcriptional Factor Enhances Phenolic Acid Production in Transgenic Roots of *Leonurus sibiricus* L. and Their Biological Activities. Mol. Biotechnol..

[120] Kumar N., Goel N. (2019). Phenolic acids: Natural versatile molecules with promising thera-peutic applications. Biotechnol. Rep. (Amst).

[121] Sun J., Peebles C.A.M. (2016). Engineering overexpression of ORCA3 and strictosidine glucosidase in *Catharanthus roseus* hairy roots increases alkaloid production. Protoplasma.

[122] Expósito O., Bonfill M., Moyano E., Onrubia M., Mirjalili M.H., Cusidó R.M., Palazón J. (2009). Biotechnological production of taxol and related taxoids: Current state and prospects. Anticancer Agents Med. Chem..

[123] Marsh S. (2006). Taxane pharmacogenetics. Per. Med..

[124] Marchev A.S., Yordanova Z.P., Georgiev M.I. (2020). Green (cell) factories for advanced production of plant secondary metabolites. Crit. Rev. Biotechnol..

[125] Zhou Z., Tan H., Li Q., Chen J., Gao S., Wang Y., Chen W., Zhang L. (2018). CRISPR/Cas9-mediated efficient targeted mutagenesis of RAS in *Salvia miltiorrhiza*. Phytochemistry.

[126] Li B., Cui G., Shen G., Zhan Z., Huang L., Chen J., Qi X. (2017). Targeted mutagenesis in the medicinal plant *Salvia miltiorrhiza*. Sci. Rep..

[127] Shkryl Y., Yugay Y., Avramenko T., Grigorchuk V., Gorpenchenko T., Grischenko O., Bulgakov V. (2021). CRISPR/Cas9-Mediated Knockout of HOS1 Reveals Its Role in the Regulation of Secondary Metabolism in *Arabidopsis thaliana*. Plants.

[128] Chun J.H., Adhikari P.B., Park S.B., Han J.Y., Choi Y.E. (2015). Production of the dammarene sapogenin (protopanaxadiol) in transgenic tobacco plants and cultured cells by heterologous expression of PgDDS and CYP716A47. Plant Cell Rep..

[129] Sitarek P., Kowalczyk T., Picot L., Michalska-Hejduk D., Bijak M., Białas A.J., Wielanek M., Śliwiński T., Skała E. (2018). Growth of *Leonurus sibiricus* L. roots with over-expression of AtPAP1 transcriptional factor in closed bioreactor, production of bioactive phenolic compounds and evaluation of their biological activity. Ind. Crops Prod..

[130] Kowalczyk T., Sitarek P., Toma M., Rijo P., Domínguez-Martín E., Falcó I., Sánchez G., Śliwiński T. (2021). Enhanced Accumulation of Betulinic Acid in Transgenic Hairy Roots of *Senna obtusifolia* Growing in the Sprinkle Bioreactor and Evaluation of Their Biological Properties in Various Biological Models. Chem. Biodivers..

[131] Ritala A., Dong L., Imseng N., Seppänen-Laakso T., Vasilev N., Van der Krol S., Rischer H., Maaheimo H., Virkki A., Brändli J. (2014). Evaluation of tobacco (*Nicotiana tabacum* L. cv. Petit Havana SR1) hairy roots for the production of geraniol, the first committed step in terpenoid indole alkaloid pathway. J. Biotechnol..

[132] Mišić D., Šiler B., Skorić M., Djurickovic M.S., Nestorović Živković J., Jovanović V., Giba Z. (2013). Secoiridoid glycosides production by *Centaurium maritimum* (L.) Fritch hairy root cultures in temporary immersion bioreactor. Process Biochem..

[133] Wang G.R., Wang H. (2019). Cell suspension culture of *Rhizoma zedoariae* in a two-stage perfusion bioreactor system for β-elemene production. Vitr. Cell. Dev. Biol. Plant.

[134] Verma P., Sharma A., Khan S.A., Shanker K., Mathur A.K. (2015). Over-expression of *Catharanthus roseus* tryptophan decarboxylase and strictosidine synthase in rol gene integrated transgenic cell suspensions of *Vinca minor*. Protoplasma.

[135] Chen X.J., Zhang X.J., Shui M., Wan J.B., Gao J.L. (2016). Anticancer Activities of Protopanaxadiol- and Protopanaxatriol-Type Ginsenosides and Their Metabolites. Evid. Based. Complement. Alternat. Med..

[136] Hussain T., Tan B., Yin Y., Blachier F., Tossou M.C., Rahu N. (2016). Oxidative Stress and Inflammation: What Polyphenols Can Do for Us?. Oxid. Med. Cell Longev..

[137] Anantharaju P.G., Gowda P.C., Vimalambike M.G. (2016). Madhunapantula SV. An overview on the role of dietary phenolics for the treatment of cancers. Nutr. J..

[138] Fulda S. (2008). Betulinic Acid for cancer treatment and prevention. Int. J. Mol. Sci..

[139] Hordyjewska A., Ostapiuk A., Horecka A., Kurzepa J. (2019). Betulin and betulinic acid: Triterpenoids derivatives with a powerful biological potential. Phytochem. Rev..

[140] Cho M., So I., Chun J.N., Jeon J.H. (2016). The antitumor effects of geraniol: Modulation of cancer hallmark pathways (Review). Int. J. Oncol..

[141] Muhamad Fadzil N.S., Sekar M., Gan S.H., Bonam S.R., Wu Y.S., Vaijanathappa J., Ravi S., Lum P.T., Dhadde S.B. (2021). Chemistry, Pharmacology and Therapeutic Potential of Swertiamarin—A Promising Natural Lead for New Drug Discovery and Development. Drug Des. Devel. Ther..

[142] Xie Q., Li F., Fang L., Liu W., Gu C. (2020). The Antitumor Efficacy of β-Elemene by Changing Tumor Inflammatory Environment and Tumor Microenvironment. Biomed. Res. Int..

[143] Zhai B., Zeng Y., Zeng Z., Zhang N., Li C., Zeng Y., You Y., Wang S., Chen X., Sui X. (2018). Drug delivery systems for elemene, its main active ingredient β-elemene, and its derivatives in cancer therapy. Int. J. Nanomed..

[144] Yin H., Sun Y.H. (2011). Vincamine-producing endophytic fungus isolated from *Vinca minor*. Phytomedicine.

[145] Al-Rashed S., Baker A., Ahmad S.S., Syed A., Bahkali A.H., Elgorban A.M., Khan M.S. (2021). Vincamine, a safe natural alkaloid, represents a novel anticancer agent. Bioorg. Chem..

[146] Burnett M.J.B., Burnett A.C. (2020). Therapeutic recombinant protein production in plants: Challenges and opportunities. Plants People Planet..

[147] Lico C., Santi L., Twyman R.M., Pezzotti M., Avesani L. (2012). The use of plants for the production of therapeutic human peptides. Plant Cell Rep..

[148] Moustafa K., Makhzoum A., Trémouillaux-Guiller J. (2016). Molecular farming on rescue of pharma industry for next generations. Crit. Rev. Biotechnol..

[149] Ma J.K.C., Drake P.M.W., Christou P. (2003). The production of recombinant pharmaceutical proteins in plants. Nat. Rev. Genet..

[150] Chen Y.C., Yeh M.K., Yeh M.K., Chen Y.C. (2018). Introductory Chapter: Biopharmaceuticals. Biopharmaceuticals.

[151] Lu R.M., Hwang Y.C., Liu I.J., Lee C.C., Tsai H.Z., Li H.J., Wu H.C. (2020). Development of therapeutic antibodies for the treatment of diseases. J. Biomed. Sci..

[152] Schillberg S., Raven N., Spiegel H., Rasche S., Buntru M. (2019). Critical analysis of the commercial potential of plants for the production of recombinant proteins. Front. Plant Sci..

[153] Dirisala V.R., Nair R.R., Srirama K., Reddy P.N., Rao K.R.S.S., Satya Sampath Kumar N., Parvatam G. (2017). Recombinant pharmaceutical protein production in plants: Unraveling the therapeutic potential of molecular pharming. Acta Physiol. Plant..

[154] Ahmad A., Pereira E.O., Conley A.J., Richman A.S., Menassa R. (2010). Green biofactories: Recombinant protein production in plants. Recent Pat. Biotechnol..

[155] Rech E., Vianna G., Murad A., Cunha N., Lacorte C., Araujo A., Brigido M., Michael W., Fontes A., Barry O. (2014). Recombinant proteins in plants. BMC Proc..

[156] Che Q., Lai H. (2015). Gene delivery into plant cells for recombinant protein production. Biomed Res. Int..

[157] Tusé D., Nandi S., McDonald K.A., Buyel J.F. (2020). The Emergency Response Capacity of Plant-Based Biopharmaceutical Manufacturing-What It Is and What It Could Be. Front. Plant Sci..

[158] Lau O.S., Sun S.S.M. (2009). Plant seeds as bioreactors for recombinant protein production. Biotechnol. Adv..

[159] Desai P.N., Shrivastava N., Padh H. (2010). Production of heterologous proteins in plants: Strategies for optimal expression. Biotechnol. Adv..

[160] Lim C.Y., Lee K.J., Oh D.B., Ko K. (2015). Effect of the developmental stage and tissue position on the expression and glycosylation of recombinant glycoprotein GA733-FcK in transgenic plants. Front. Plant Sci..

[161] Kang Y., Shin Y.K., Park S.W., Ko K. (2016). Effect of nitrogen deficiency on recombinant protein production and dimerization and growth in transgenic plants. Hortic. Environ. Biotechnol..

[162] Lu Z., Lee K.J., Shao Y., Lee J.H., So Y., Choo Y.K., Oh D.B., Hwang K.A., Oh S.H., Han Y.S. (2012). Expression of GA733-Fc fusion protein as a vaccine candidate for colorectal cancer in transgenic plants. J. Biomed. Biotechnol..

[163] Jez J., Castilho A., Grass J., Vorauer-Uhl K., Sterovsky T., Altmann F., Steinkellner H. (2013). Expression of functionally active sialylated human erythropoietin in plants. Biotechnol. J..

[164] Thomas D.R., Walmsley A.M. (2014). Improved expression of recombinant plant-made hEGF. Plant Cell Rep..

[165] Luchakivskaya Y., Kishchenko O., Gerasymenko I., Olevinskaya Z., Simonenko Y., Spivak M., Kuchuk M. (2011). High-level expression of human interferon alpha-2b in transgenic carrot (*Daucus carota* L.) plants. Plant Cell Rep..

[166] Mercx S., Smargiasso N., Chaumont F., De Pauw E., Boutry M., Navarre C. (2017). Inactivation of the β(1,2)-xylosyltransferase and the α(1,3)-fucosyltransferase genes in *Nicotiana tabacum* BY-2 Cells by a Multiplex CRISPR/Cas9 Strategy Results in Glycoproteins without Plant-Specific Glycans. Front. Plant Sci..

[167] Matsuo K. (2021). CRISPR/Cas9-mediated knockout of the DCL2 and DCL4 genes in *Nicotiana benthamiana* and its productivity of recombinant proteins. Plant Cell Rep..

[168] Macharoen K., McDonald K.A., Nandi S. (2020). A method to simplify bioreactor processing for recombinant protein production in rice cell suspension cultures. MethodsX.

[169] Macharoen K., Du M., Jung S., McDonald K.A., Nandi S. (2021). Production of recombinant butyrylcholinesterase from transgenic rice cell suspension cultures in a pilot-scale bioreactor. Biotechnol. Bioeng..

[170] Macharoen K., Li Q., Márquez-Escobar V.A., Corbin J.M., Lebrilla C.B., Nandi S., McDonald K.A. (2020). Effects of kifunensine on production and n-glycosylation modification of butyrylcholinesterase in a transgenic rice cell culture bioreactor. Int. J. Mol. Sci..

[171] Corbin J.M., Hashimoto B.I., Karuppanan K., Kyser Z.R., Wu L., Roberts B.A., Noe A.R., Rodriguez R.L., McDonald K.A., Nandi S. (2016). Semicontinuous bioreactor production of recombinant butyrylcholinesterase in transgenic rice cell suspension cultures. Front. Plant Sci..

[172] Kwon J.Y., Cheon S.H., Nam H.J., Choi H.Y., Kim D. (2013). Process characterization of hCTLA4Ig production in transgenic rice cell cultures using a 3-L bioreactor. Appl. Biochem. Biotechnol..

[173] Kwon J.Y., Yang Y.S., Cheon S.H., Nam H.J., Jin G.H., Kim D. (2013). Bioreactor engineering using disposable technology for enhanced production of hCTLA4Ig in transgenic rice cell cultures. Biotechnol. Bioeng..

[174] Park C.I., Lee S.J., Kang S.H., Jung H.S., Kim D., Lim S.M. (2010). Fed-batch cultivation of transgenic rice cells for the production of hCTLA4Ig using concentrated amino acids. Process Biochem..

[175] Myoung H.J., Choi H.Y., Nam H.J., Kim D.I. (2015). In situ Recovery of hGM-CSF in Transgenic Rice Cell Suspension Cultures. KSBB J..

[176] López E.G., Ramírez E.G.R., Gúzman O.G., Calva G.C., Ariza-Castolo A., Pérez-Vargas J., Rodríguez H.G.M. (2014). MALDI-TOF characterization of hGH1 produced by hairy root cultures of *Brassica oleracea* var. *italica* grown in an airlift with mesh bioreactor. Biotechnol. Prog..

[177] Michoux F., Ahmad N., Hennig A., Nixon P.J., Warzecha H. (2013). Production of leafy biomass using temporary immersion bioreactors: An alternative platform to express proteins in transplastomic plants with drastic phenotypes. Planta.

[178] Liu Y.K., Huang L.F., Ho S.L., Liao C.Y., Liu H.Y., Lai Y.H., Yu S.M., Lu C.A. (2012). Production of mouse granulocyte-macrophage colony-stimulating factor by gateway technology and transgenic rice cell culture. Biotechnol. Bioeng..

[179] Raven N., Rasche S., Kuehn C., Anderlei T., Klöckner W., Schuster F., Henquet M., Bosch D., Büchs J., Fischer R. (2015). Scaled-up manufacturing of recombinant antibodies produced by plant cells in a 200-L orbitally-shaken disposable bioreactor. Biotechnol. Bioeng..

[180] Macharoen K., McDonald K.A., Nandi S. (2020). Simplified bioreactor processes for recombinant butyrylcholinesterase production in transgenic rice cell suspension cultures. Biochem. Eng. J..

[181] Singh A., Srivastava S., Chouksey A., Panwar B.S., Verma P.C., Roy S., Singh P.K., Saxena G., Tuli R. (2015). Expression of Rabies Glycoprotein and Ricin Toxin B Chain (RGP–RTB) Fusion Protein in Tomato Hairy Roots: A Step Towards Oral Vaccination for Rabies. Mol. Biotechnol..

[182] Michoux F., Ahmad N., Mccarthy J., Nixon P.J. (2011). Contained and high-level production of recombinant protein in plant chloroplasts using a temporary immersion bioreactor. Plant Biotechnol, J..

[183] Liu Y.K., Li Y.T., Lu C.F., Huang L.F. (2015). Enhancement of recombinant human serum albumin in transgenic rice cell culture system by cultivation strategy. N. Biotechnol..

[184] Reuter L.J., Bailey M.J., Joensuu J.J., Ritala A. (2014). Scale-up of hydrophobin-assisted recombinant protein production in tobacco BY-2 suspension cells. Plant Biotechnol. J..

[185] Top O., Parsons J., Bohlender L.L., Michelfelder S., Kopp P., Busch-Steenberg C., Hoernstein S.N.W., Zipfel P.F., Häffner K., Reski R. (2019). Recombinant production of mfhr1, a novel synthetic multitarget complement inhibitor, in moss bioreactors. Front. Plant Sci..

[186] Kim S.R., Sim J.S., Ajjappala H., Kim Y.H., Hahn B.S. (2012). Expression and large-scale production of the biochemically active human tissue-plasminogen activator in hairy roots of Oriental melon (*Cucumis melo*). J. Biosci. Bioeng..

[187] Darvesh S., Hopkins D.A., Geula C. (2003). Neurobiology of butyrylcholinesterase. Nat. Rev. Neurosci..

[188] Nyberg F., Hallberg M. (2013). Growth hormone and cognitive function. Nat. Rev. Endocrinol..

[189] Ranke M.B., Lindberg A. (2009). Predicting growth in response to growth hormone treatment. Growth Horm. IGF Res..

[190] Buchbinder E., Stephen Hodi F. (2015). Cytotoxic T lymphocyte antigen-4 and immune checkpoint blockade. J. Clin. Investig..

[191] Waterhouse P., Penninger J.M., Timms E., Wakeham A., Shahinian A., Lee K.P., Thompson C.B., Griesser H., Mak T.W. (1995). Lymphoproliferative disorders with early lethality in mice deficient in Ctla-4. Science.

[192] Shi Y., Liu C.H., Roberts A.I., Das J., Xu G., Ren G., Zhang Y., Zhang L., Zeng R.Y., Tan H.S.W. (2006). Granulocyte-macrophage colony-stimulating factor (GM-CSF) and T-cell responses: What we do and don’t know. Cell Res..

[193] Morrison T.B., Weis J.H., Weis J.J. (1997). *Borrelia burgdorferi* Outer Surface Protein a (OspA) Activates and Primes Human Neutrophils. J. Immunol..

[194] Lambert J.M., Goldmacher V.S., Collinson A.R., Nadler L.M., Bllattler W.A. (1991). An Immunotoxin Prepared with Blocked Ricin: A Natural Plant Toxin Adapted for Therapeutic Use. Cancer Res..

[195] Pellizzari R., Rossetto O., Schiavo G., Montecucco C. (1999). Tetanus and botulinum neurotoxins: Mechanism of action and therapeutic uses. Philos. Trans. R. Soc. Lond. B Biol. Sci..

[196] Caraceni P., Tufoni M., Bonavita M.E. (2013). Clinical use of albumin. Blood Transfus..

[197] Mendez C.M., McClain C.J., Marsano L.S. (2005). Albumin therapy in clinical practice. Nutr. Clin. Pract..

[198] Gravanis I., Tsirka S.E. (2008). Tissue-type plasminogen activator as a therapeutic target in stroke. Expert Opin. Ther. Targets.

[199] Wösten H.A.B., Scholtmeijer K. (2015). Applications of hydrophobins: Current state and perspectives. Appl. Microbiol. Biotechnol..

[200] Häkkinen S.T., Moyano E., Cusidó R.M., Oksman-Caldentey K.M. (2016). Exploring the metabolic stability of engineered hairy roots after 16 years maintenance. Front. Plant Sci..

[201] Mardanova E.S., Blokhina E.A., Tsybalova L.M., Peyret H., Lomonossoff G.P., Ravin N.V. (2017). Efficient transient expression of recombinant proteins in plants by the novel pEff vector based on the genome of potato virus X. Front. Plant Sci..

[202] Martí M., Diretto G., Aragonés V., Frusciante S., Ahrazem O., Gómez-Gómez L., Daròs J.A. (2020). Efficient production of saffron crocins and picrocrocin in Nicotiana benthamiana using a virus-driven system. Metab. Eng..

[203] Knott G.J., Doudna J.A. (2018). CRISPR-Cas guides the future of genetic engineering. Science..

[204] Zhu H., Li C., Gao C. (2020). Applications of CRISPR-Cas in agriculture and plant biotechnology. Nat. Rev. Mol. Cell Biol..

